# Characterization of Haartman Institute snake virus-1 (HISV-1) and HISV-like viruses—The representatives of genus *Hartmanivirus*, family *Arenaviridae*

**DOI:** 10.1371/journal.ppat.1007415

**Published:** 2018-11-14

**Authors:** Jussi Hepojoki, Satu Hepojoki, Teemu Smura, Leonóra Szirovicza, Eva Dervas, Barbara Prähauser, Lisbeth Nufer, Elisabeth M. Schraner, Olli Vapalahti, Anja Kipar, Udo Hetzel

**Affiliations:** 1 University of Helsinki, Faculty of Medicine, Medicum, Department of Virology, Helsinki, Finland; 2 Institute of Veterinary Pathology, Vetsuisse Faculty, University of Zurich, Zurich, Switzerland; 3 Boid Inclusion Body Disease Group, Institute of Veterinary Pathology, University of Zurich, Zurich, Switzerland; 4 Institutes of Veterinary Anatomy and Virology, Vetsuisse Faculty, University of Zurich, Zurich, Switzerland; 5 University of Helsinki, Faculty of Veterinary Medicine, Department of Veterinary Biosciences, Helsinki, Finland; 6 Department of Virology and Immunology, HUSLAB, Helsinki University Hospital, Helsinki, Finland; Division of Clinical Research, UNITED STATES

## Abstract

The family *Arenaviridae* comprises three genera, *Mammarenavirus*, *Reptarenavirus* and the most recently added *Hartmanivirus*. Arenaviruses have a bisegmented genome with ambisense coding strategy. For mammarenaviruses and reptarenaviruses the L segment encodes the Z protein (ZP) and the RNA-dependent RNA polymerase, and the S segment encodes the glycoprotein precursor and the nucleoprotein. Herein we report the full length genome and characterization of Haartman Institute snake virus-1 (HISV-1), the putative type species of hartmaniviruses. The L segment of HISV-1 lacks an open-reading frame for ZP, and our analysis of purified HISV-1 particles by SDS-PAGE and electron microscopy further support the lack of ZP. Since we originally identified HISV-1 in co-infection with a reptarenavirus, one could hypothesize that co-infecting reptarenavirus provides the ZP to complement HISV-1. However, we observed that co-infection does not markedly affect the amount of hartmanivirus or reptarenavirus RNA released from infected cells *in vitro*, indicating that HISV-1 does not benefit from reptarenavirus ZP. Furthermore, we succeeded in generating a pure HISV-1 isolate showing the virus to replicate without ZP. Immunofluorescence and ultrastructural studies demonstrate that, unlike reptarenaviruses, HISV-1 does not produce the intracellular inclusion bodies typical for the reptarenavirus-induced boid inclusion body disease (BIBD). While we observed HISV-1 to be slightly cytopathic for cultured boid cells, the histological and immunohistological investigation of HISV-positive snakes showed no evidence of a pathological effect. The histological analyses also revealed that hartmaniviruses, unlike reptarenaviruses, have a limited tissue tropism. By nucleic acid sequencing, *de novo* genome assembly, and phylogenetic analyses we identified additional four hartmanivirus species. Finally, we screened 71 individuals from a collection of snakes with BIBD by RT-PCR and found 44 to carry hartmaniviruses. These findings suggest that harmaniviruses are common in captive snake populations, but their relevance and pathogenic potential needs yet to be revealed.

## Author summary

From the 1930s to 2015 arenaviruses were known as mainly rodent-borne viruses, which occasionally infect humans, causing a severe disease. After isolation of novel arenaviruses from snakes, the family *Arenaviridae* now comprises three genera, *Mammarenavirus*, *Reptarenavirus*, and *Hartmanivirus*. Here we characterize a pure isolate of the putative hartmanivirus type species, Haartman Institute snake virus-1 (HISV-1) and report three new hartmanivirus species: Old Schoolhouse virus, Dante Muikkunen virus and Veterinary Pathology Zurich virus. The genomes of all these hartmaniviruses lack the matrix protein found in other arenaviruses, and using HISV-1 as a model we demonstrate that hartmaniviruses can nonetheless form infectious particles. Although HISV-1 induces cytopathic changes in cell culture, there was no evidence of a cytopathogenic effect in infected snakes, where hartmaniviruses have a restricted cell tropism. We further identified hartmanivirus infection in over 60% of snakes in a single breeding collection, indicating that hartmaniviruses are relatively common in boid snakes. Several animals of the collection studied were diagnosed with BIBD, and it is not clear whether hartmaniviruses play a role in the development of BIBD in reptarenavirus-infected snakes. The co-infection with viruses from two genera of the same virus family provides an interesting model for future studies.

## Introduction

The first member of the family *Arenaviridae*, lymphocytic choriomeningitis virus (LCMV), was identified and isolated already in the 1930s [1]. During the following four decades several novel members of the family were identified including human pathogens such as Junin (JUNV), Machupo (MACV), and Lassa (LASV) viruses [1]. For decades, arenaviruses were known as rodent-borne viruses with the exception of Tacaribe virus (TCRV), which was isolated from a bat [1]. In the early 2010s, three independent groups identified novel arenaviruses as the potential causative agents for boid inclusion body disease (BIBD) [2–6]. BIBD is characterized by intracellular cytoplasmic inclusion bodies (IB) within almost all cell types of affected snakes [4, 7, 8]. The IB mainly (or solely) consist of arenavirus nucleoprotein (NP) [4, 8], and BIBD was recently successfully reproduced by experimental reptarenavirus infection [9]. The identification of the “snake arenaviruses” prompted the establishment of two new genera, *Mammarenavirus* and *Reptarenavirus*, within the family *Arenaviridae* [1]. Snakes with BIBD often, if not always, carry L and S segments of several reptarenavirus species [10, 11]. Furthermore, infected snakes usually harbor more L than S segments, which significantly hampers the taxonomic classification of reptarenaviruses [10–12]. The International Committee on Taxonomy of Viruses (ICTV) *Arenaviridae* study group has recommended that the PAirwise Sequence Comparison (PASC, available at (https://www.ncbi.nlm.nih.gov/sutils/pasc/viridty.cgi?textpage=overview) tool should be used for genus and species determination [1]. The PASC tool classifies arenaviruses to the same genus if the nucleotide sequence identity in the S segment is >29–40% and >30–35% in the L segment [1, 13]. When analyzing some of the virus isolates of our first paper on BIBD [4], we found a virus genome with coding strategy similar to arenaviruses and named the isolate Haartman Institute snake virus-1 (HISV-1) [10]. Analysis of the HISV-1 genome with the PASC tool showed that HISV-1 represents a novel arenavirus genus, and in 2018 the third genus, *Hartmanivirus*, was established in the family *Arenaviridae* [14].

Arenaviruses are RNA viruses with a single-stranded, bisegmented, negative-sense RNA genome and an ambisense coding strategy [1]. The large (L) genome segment encodes matrix/Z protein (ZP) and RNA-dependent RNA polymerase (RdRp) and the small (S) segment encodes glycoprotein precursor (GPC) and nucleoprotein (NP)[1]. Arenaviruses replicate in the cytoplasm of the infected cells, the genome replication and transcription requires both RdRp and NP [15]. Initially, the ZP was also thought to contribute to the latter processes [16] but later studies have demonstrated that ZP rather acts to suppress both [15, 17]. All structural proteins of arenaviruses have essential roles in the arenavirus life cycle: RdRp is required for genome replication, GPC for spike formation to gain cell entry, NP for genome packaging and replication, and ZP for budding and regulation of replication [15, 18]. Additionally, the NPs of all mammarenaviruses but TCRV inhibit type I interferon (IFN-I) induction [19] at multiple steps of the signaling pathway [20]. Likewise, the ZPs of mammarenaviruses that are pathogenic in humans inhibit IFN-I signaling by targeting RIG-I and MDA5 [20]. The ZPs also interact with cellular components such as PML (promyelocytic leukemia protein), eIF4E (eukaryotic translation initiation factor 4E), and the ESCRT (endosomal sorting complexes required for transport) system required for budding [20].

When assembling the genome of HISV-1 we observed that the L segment lacks an open-reading frame (ORF) for the ZP [10]. At that point, we did not have a pure HISV-1 isolate and we could thus neither confirm the latter finding nor could we investigate whether HISV-1 would survive without co-infecting reptarenavirus(es). Herein, we report the isolation and characterization of a pure HISV-1 cell culture isolate demonstrating that infectious virions are produced despite the lack of ZP. We also identified three additional hartmanivirus species and provide the complete coding regions for their genomes along with a number of nearly complete reptarenavirus genome segments. We identified hartmaniviruses in snakes with BIBD, but could also detect hartmanivirus infection in apparently healthy snakes, suggesting that these viruses are not directly linked to BIBD pathogenesis. Even though we could not associate hartmanivirus infection with pathological changes *in vivo*, we observed cytopathic effects of HISV-1 infection *in vitro*.

## Results

### Generation of a pure HISV-1 isolate and genome sequencing

Previously we used next-generation sequencing (NGS) for the characterization of reptarenavirus isolates [4]. This led to the identification of HISV-1, a putative representative for novel arenavirus genus, in addition to several reptarenavirus S and L segments [10]. Even though we had fairly high coverage of the S (11–27092 fold) and L (357–4493 fold) segments of HISV-1, we were unsure whether these represented the full length segments since the L segment comprised an ORF for the RdRp [10], but the ORF for the ZP found in other arenaviruses was missing. Since the original HISV-1 preparation contained also a reptarenavirus (UHV-2, University of Helsinki virus-2), we used serial dilution to obtain single virus isolates of both UHV-2 and HISV-1 (S1 Fig). Successful production of a clean HISV-1 isolate indicated that even though an ORF for the ZP, which functions as the matrix protein, cannot be found in the L segment, HISV-1 is able to replicate without a co-infecting reptarenavirus. For most subsequent comparative experiments we used UHV-2 as the reference reptarenavirus, since it was the co-infecting reptarenavirus of the original HISV-1 isolate. For a few experiments we used UGV-1 (University of Giessen virus-1) instead, as this is a reptarenavirus grows to high titers in our cell culture model. For the sequence analyses we chose to use the type species of each arenavirus genera.

To confirm the lack of an ORF for the ZP in the L segment of HISV-1, we isolated RNA from a batch of purified HISV-1 and sequenced the ends of the S and L segments using T4 RNA ligase to generate cyclic RNAs. The latter then served as templates for RT-PCR over the genome ends (S2 Fig) and yielded several sequences that indeed covered the genome ends of both the S and L segment. In addition to the S and L segment specific primer pairs, we also successfully applied primer combinations, i.e. L segment forward-S segment reverse and S segment forward-L segment reverse, in RT-PCRs, which suggested that T4 RNA ligation also produced S and L segment chimeras. This was confirmed by sequencing. The genome end sequencing then confirmed that the L segment of HISV-1 indeed lacks the matrix protein/ZP found in other arenaviruses. Subsequent NGS and *de novo* genome assembly for the purified HISV-1 preparation did not identify additional genome segments. The consensus sequence of the S segment revealed a nucleotide insertion (a stretch of 7 instead of 6 adenines at 575–581) in the GP1 ORF of our original submission [10], which led to incomplete “*in silico*” translation of the GPC.

### Comparison of the HISV-1 genome and proteome with those of other arenaviruses

**Fig 1:**
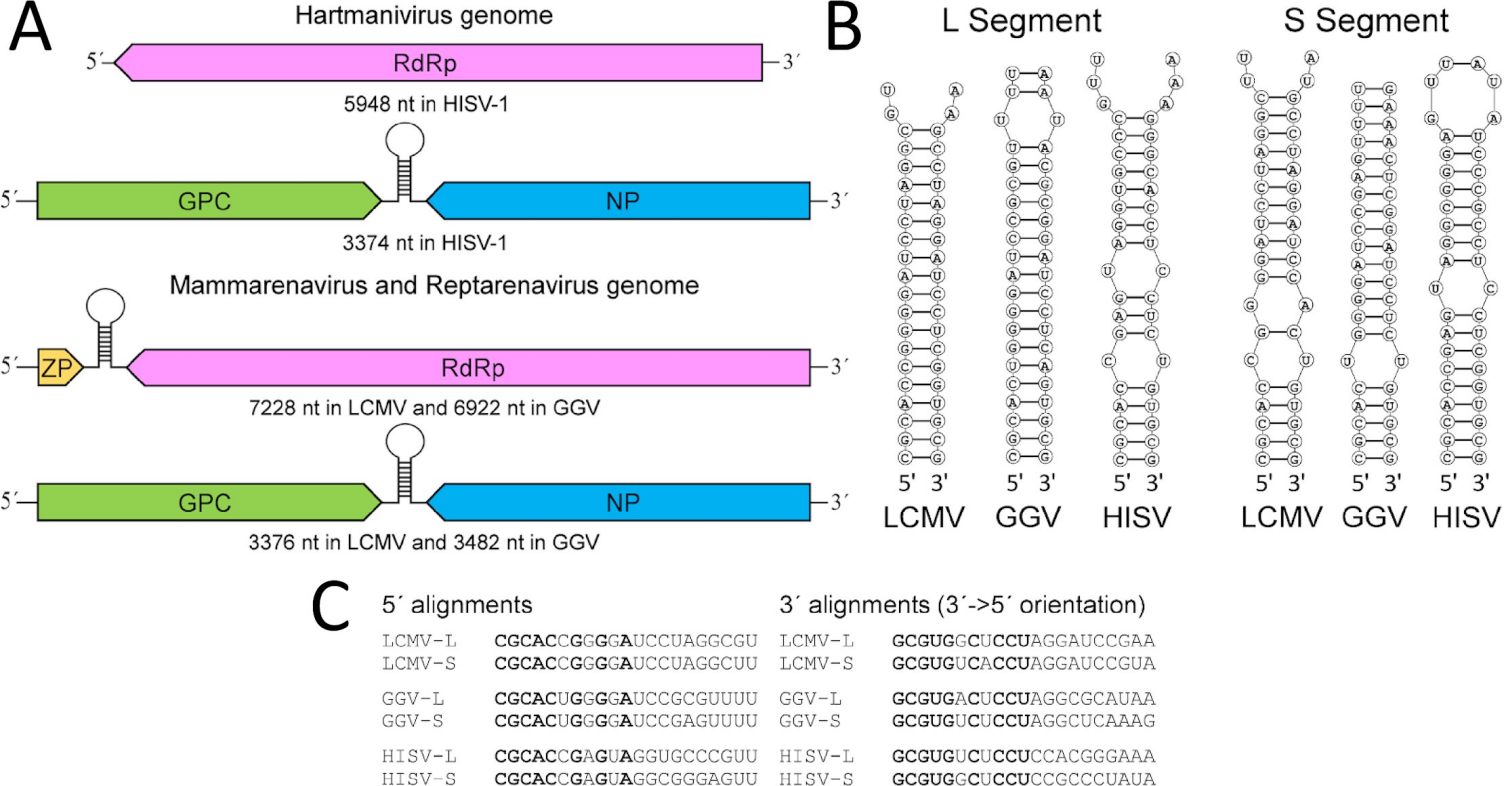
The genome organization of the family *Arenaviridae* members. **A)** The genome segments with respective sizes for the type species of the genera *Hartmanivirus* (HISV-1), Mammarenavirus (LCMV) and *Reptarenavirus* (GGV). The ORFs (ZP = Z protein, RdRp = RNA-dependent RNA polymerase, GPC = glycoprotein precursor, NP = nucleoprotein) are shown as arrows to demonstrate the direction of translation respective to genome orientation. **B)** Panhandle structures formed by the genome ends as predicted using DuplexFold Web Server of RNA structure (available at https://rna.urmc.rochester.edu/RNAstructureWeb/Servers/DuplexFold/DuplexFold.html) for LCMV, GGV, and HISV-1. **C)** Alignments of the 5´ and 3´ (shown in 5´ = >3´ orientation) genome ends for L and S segments of LCMV, GGV, and HISV-1. The nucleotide residues conserved throughout the L and S segments of family *Arenaviridae* genera are shown in bold.

Obtaining the full length genome segments of HISV-1 allowed us to compare the genomes of the three arenavirus genera, *Mammarenavirus*, *Reptarenavirus*, and *Hartmanivirus*. Since this is the first time that full length genome segments of all arenavirus genera are available, we decided to perform a bioinformatics-based comparison of their genomes and proteomes, and selected the type species of each genus for these “*in silico*” comparisons. The S segments of all three genera, schematically depicted in Fig 1A, are identical in their coding strategy and similar in size. However, while the L segments of mammarenaviruses and reptarenaviruses share the same coding strategy and are similar in size, the L segment of hartmaniviruses lacks the ZP ORF (Fig 1A).

As shown in Fig 1B, the genome ends–represented by 21 terminal nucleotides–of all arenavirus species have the ability to form a panhandle structure. The panhandles of LCMV and Golden Gate virus (GGV) L segments comprise 18 consecutive complementary nucleotides, while the corresponding region in the HISV-1 L segment contains two non-paired nucleotides. Comparison of the genome segment ends revealed a conserved CGCACxGxGxA motif at the 5´ end of mammarena-, reptarena-, and hartmanivirus S and L segments (shown in bold in Fig 1C). Similarly, the 3´ends show conserved nucleotides, GCGUGxCxCCU (shown in bold in Fig 1C), complementary to those found at the 5´ end with the underlined residue making an exception. While the predicted panhandle structures may differ even between viruses of the same species, the overall panhandle structure is maintained throughout arenavirus genera by the aforementioned conserved nucleotides. The terminal complementarity of the RNA is essential for replication and transcription [21] which, at least partially, may explain the conservation of the segment ends throughout the family. The non-conserved sites at both ends are speculated not to contribute to sequence-specific interaction with the RdRp [22], thus explaining the observed variation at these sites.

The proteomes and the amino acid identities between the corresponding proteins (based on MAFFT alignment) of the three genera are presented in S2 Table. The major difference in the proteomes is the lack of ZP in hartmaniviruses. While the ZPs of reptarenaviruses and mammarenaviruses share only 16% amino acid identity, their functions are assumed to be similar [2]. Interestingly, reptarenavirus ZPs have an N-terminal transmembrane helix (TM) [2, 4], whereas the N-terminus of mammarenaviruses is myristoylated [2]. The structural proteins of HISV-1 are all 20 to 23% identical to their LCMV and GGV counterparts. The RdRp and NP of GGV are slightly closer to LCMV (28% and 32% identical) than to HISV (20% and 20% identical). However, the GPCs of HISV-1 and LCMV (23% identity) are more similar than the GPCs of LCMV and GGV (16% identity). See S2 Table for more detail.

**Fig 2:**
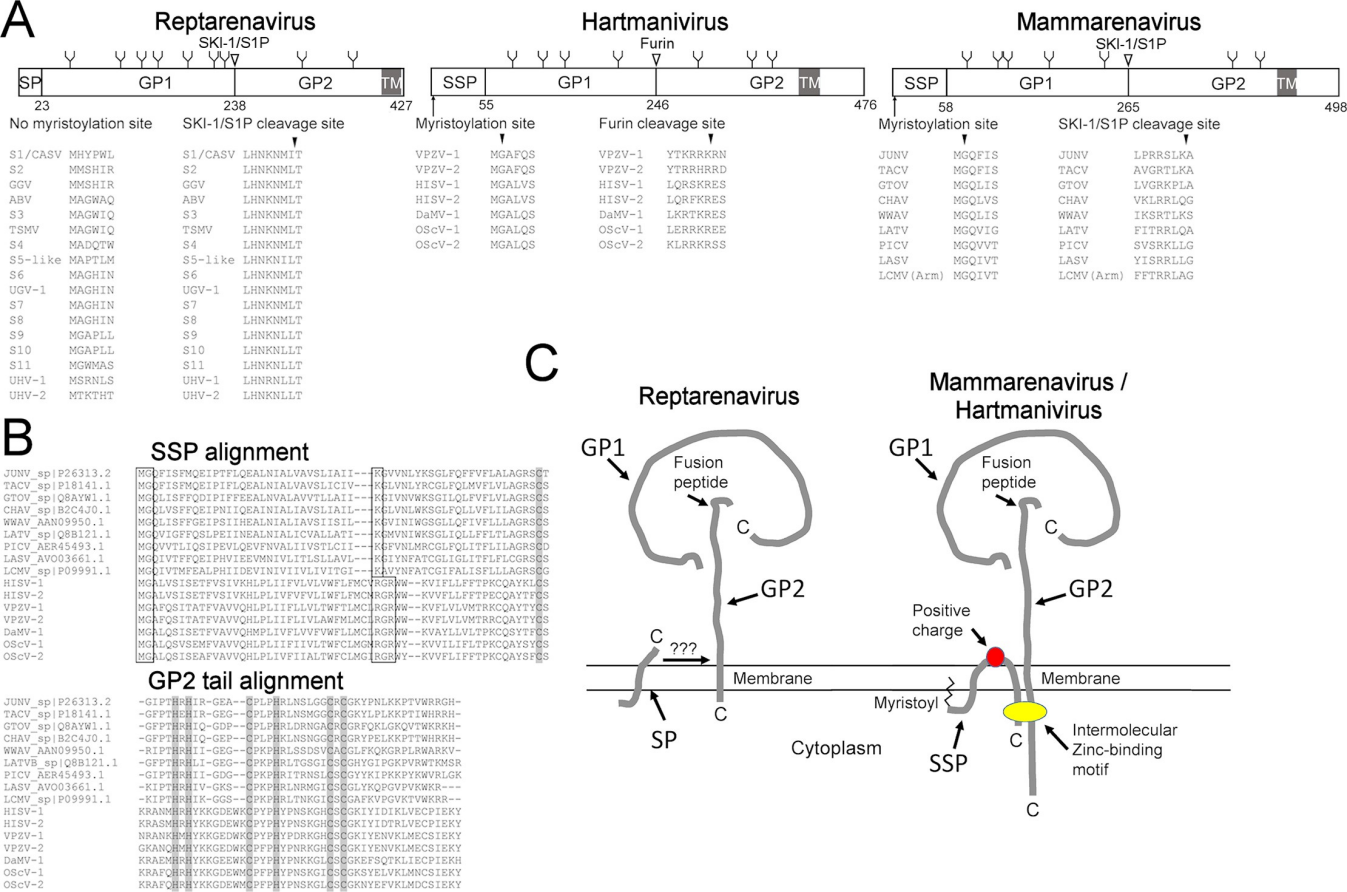
Comparison of the GPC ORF between the three arenavirus genera. **A)** The GPCs of the genera mammarenavirus, reptarenavirus, and hartmanivirus as exemplified by the respective type species LCMV, GGV and HISV-1. The highlighted features are: cleavage sites (presented by vertical line and number), glycosylation sites (presented by Y-shape) and transmembrane region (TM, presented by grey shading) are based on LCMV, GGV, and HISV-1 sequences. The abbreviations are: SSP = stable signal peptide, SP = signal peptide, GP1 = glycoprotein 1, GP2 = glycoprotein 2, TM = transmembrane. The myristoylation site of mammarenavirus and hartmanivirus SSP is shown by an arrow and below is an alignment of the site in other species of the genus. SKI-1/S1P cleavage site in mammarenavirus and reptarenavirus GPC is depicted by an arrow at around the center of GPC, and below is an alignment around the cleavage site for other species of the genus. For hartmaniviruses furin performs the GP1-GP2 cleavage instead of SKI-1/S1P. **B)** Alignment of mammarena- and hartmanivirus SSP and GP2, the residues essential for SSP-GP2 intermolecular zinc-binding motif are depicted by grey highlighting. The conserved myristoylation in the SSP is highlighted by a box, as well as the positive charge at around the center of SSP. **C)** A schematic representation of the glycoprotein complex as inspired by the illustration of Nunberg and York[17].

The GPC of each arenavirus genera are schematically presented in Fig 2A. By prediction the GPCs contain several N-glycosylation sites: 7 in HISV-1 (4 in GP1 and 3 in GP2) and LCMV (5 in GP1 and 2 in GP2) and 9 in GGV (7 in GP1 and 2 in GP2) (Fig 2A). The cleavage between GP1 and GP2 is mediated by subtilisin-kexin isozyme-1/site-1 protease (SKI-1/S1P) for mammarenaviruses (Fig 2A). Using ELM (eukaryotic linear motif resource, http://elm.eu.org/, [23]) we identified a potential SKI-1/S1P cleavage site in GGV GPC, and the GPC alignment of known reptarenavirus species suggests that the site is conserved (Fig 2A). For HISV-1 we could not detect a SKI-1/S1P cleavage site but rather identified a potent furin cleavage site in the same region. By comparing the region to GPCs of the other hartmanivirus species (described later in the manuscript) we could show that the furin cleavage site is preserved among the hartmaniviruses found thus far (Fig 2A).

A more thorough investigation of the GPCs indeed shows similarities between HISV-1 and LCMV. To begin with, both HISV-1 and LCMV have long (55 and 58 residues, respectively) signal peptides (SP) that by prediction are myristoylated at position 2, while the SP of GGV is only 23 residues and lacks the myristoylation site (Fig 2A). For mammarenaviruses the SP remains in the virion and is known as a stable signal peptide (SSP) that interacts with GP2 via an intermolecular zinc-binding motif [24] (Fig 2C). Sequence comparison between mammarenavirus and hartmanivirus SSPs and GP2s (Fig 2B) shows conservation at the histidine and cysteine residues required for the SSP-GP2 interaction. Additionally, the GP2s of both mammarenaviruses and hartmaniviruses have a relatively long cytoplasmic tails (48 and 42 residues respectively) while the GP2 tail of reptarenaviruses only comprises a few (two in GGV) residues (Fig 2A and 2B). The above suggests that there might be differences in the organization of the spikes complex between reptarenaviruses and the other two arenavirus genera. Of note, the GP2 is the most conserved reptarenavirus protein, sharing 68–99% sequence identity between all the known species and 87–99% identity when CASV and UHV-1 are excluded. The GP2s of both mammarenaviruses and hartmaniviruses appear to be more variable.

### *In vitro* characterization of HISV-1 infection

**Fig 3:**
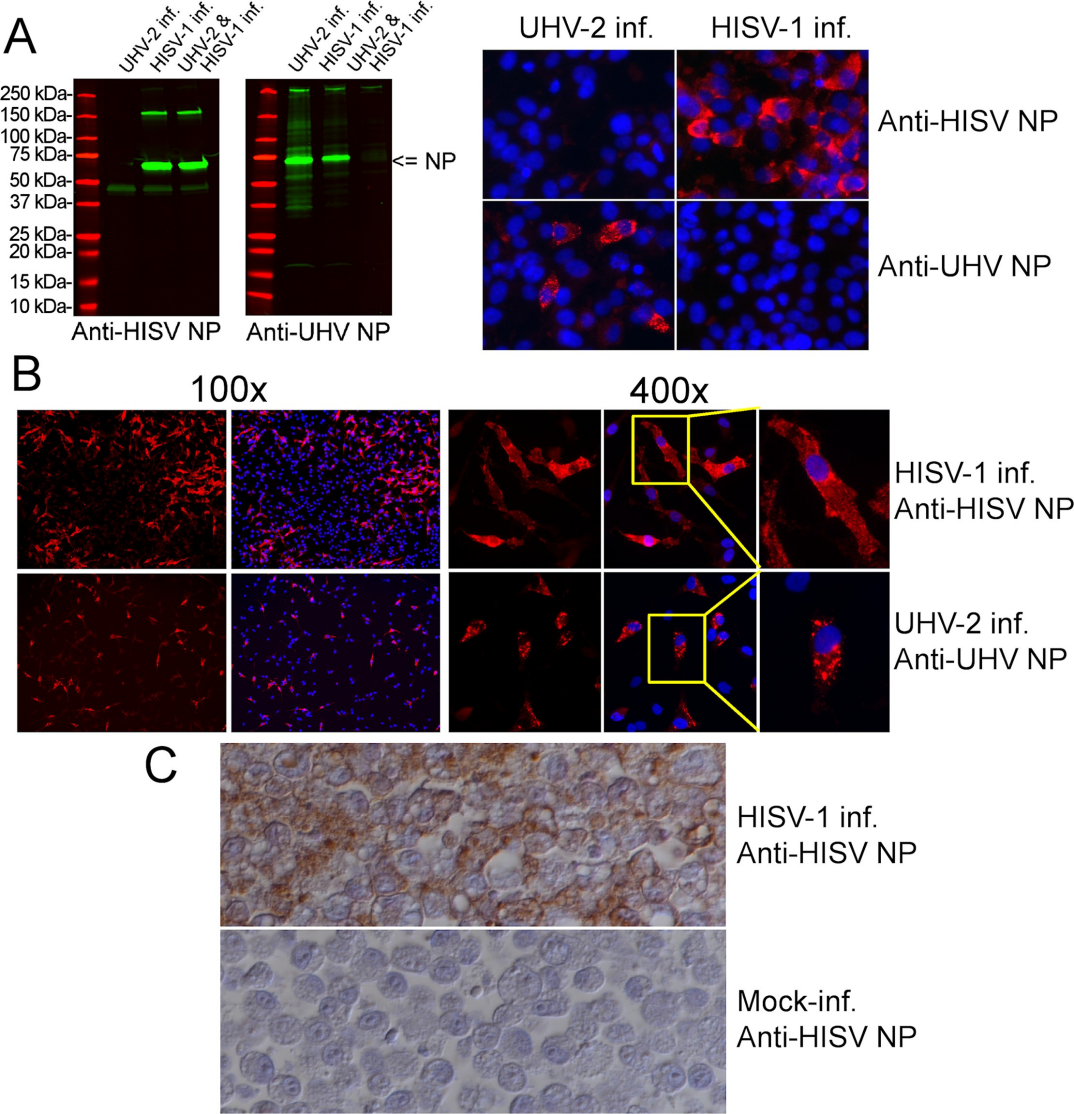
Western blot (WB), immunofluorescence (IF), and immunohistology (IH) of cultured cells using antiserum against C-terminal portion of HISV-1 (anti-HISV NP) or UHV-1 (anti-UHV NP) NP. **A)** The panels on right show WBs comparing I/1Ki cells infected with UHV-2 or HISV-1 alone to I/1Ki cells co-infected with UHV-2 and HISV-1. The molecular weight marker (Precision Plus Protein All Blue Prestained Protein Standards, Bio-Rad) is shown in red, and the staining with anti-HISV NP (left) and anti-UHV NP (right) is shown in green. The figure was obtained using Odyssey infrared imaging system (LI-COR). The panels on left show IF staining of I/1Ki cells infected with UHV-2 alone or HISV-1 alone using anti-HISV NP (top panels) and anti-UHV NP (bottom panels). The cells were stained at two days post infection (dpi). The red channel shows NP staining and the blue channel nuclei (DAPI staining). The figures were captured at 400x magnification. **B)** I/1Ki cells infected with HISV-1 or UHV-2 IF stained at 2 dpi. The top panels show HISV-1 infected cells stained with anti-HISV NP and the bottom panels show UHV-2 infected cells stained with anti-UHV NP. The red channel shows NP staining and the blue channel nuclei (Hoechst staining). The panels on left side were captured at 100x and the panels on right side at 400x magnification. The yellow square indicates the individual cells shown at higher magnification on the rightmost panels. **C)** IH of non-infected and HISV-1 infected I/1Ki cells. The HISV-1 infected cells were collected and fixed at 6 dpi, the figures were captured at 400x magnification.

To monitor and characterize HISV-1 infection in cell culture, we produced an antiserum against the HISV-1 NP which we refer to as anti-HISV NP antiserum throughout the manuscript. We used the C-terminal half of the NP as the antigen, since this strategy had produced a broadly reactive anti-UHV-1 NP antiserum [10] referred to as anti-UHV NP antiserum throughout the manuscript. To test the anti-HISV NP antiserum, we performed infection and co-infection experiments with UHV-2 and HISV-1 on cultured boid kidney cells (I/1Ki), and analyzed the infected cells by western blotting, immunofluorescence (IF) staining, and immunohistology (IH) (Fig 3). The western blot shows that the anti-HISV NP antiserum does not cross-react with UHV-2 NP, and vice versa (Fig 3A). The IF staining of infected cells concurs with the western blotting results, and also indicates that neither of the antisera reacts with cellular proteins at the concentrations applied (Fig 3A). A comparison of the IF staining patterns in infected cells showed that HISV-1 infection produces large fluorescent foci (i.e. infection of several cells in close proximity to each other), whereas UHV-2 infection resulted in scattered individual positive cells (100x magnification in Fig 3B). At a higher magnification, UHV-2 infected cells were found to exhibit the punctate NP staining pattern typical for reptarenavirus infected cells with inclusion bodies (IB)[4, 25], whereas HISV-1 NP appeared to be more diffusely distributed in the cells (400x magnification in Fig 3B). A similar staining pattern was seen in HISV-1 infected I/1Ki cell pellets, indicating that the antiserum is suitable for immunohistology (IH) (Fig 3C).

**Fig 4:**
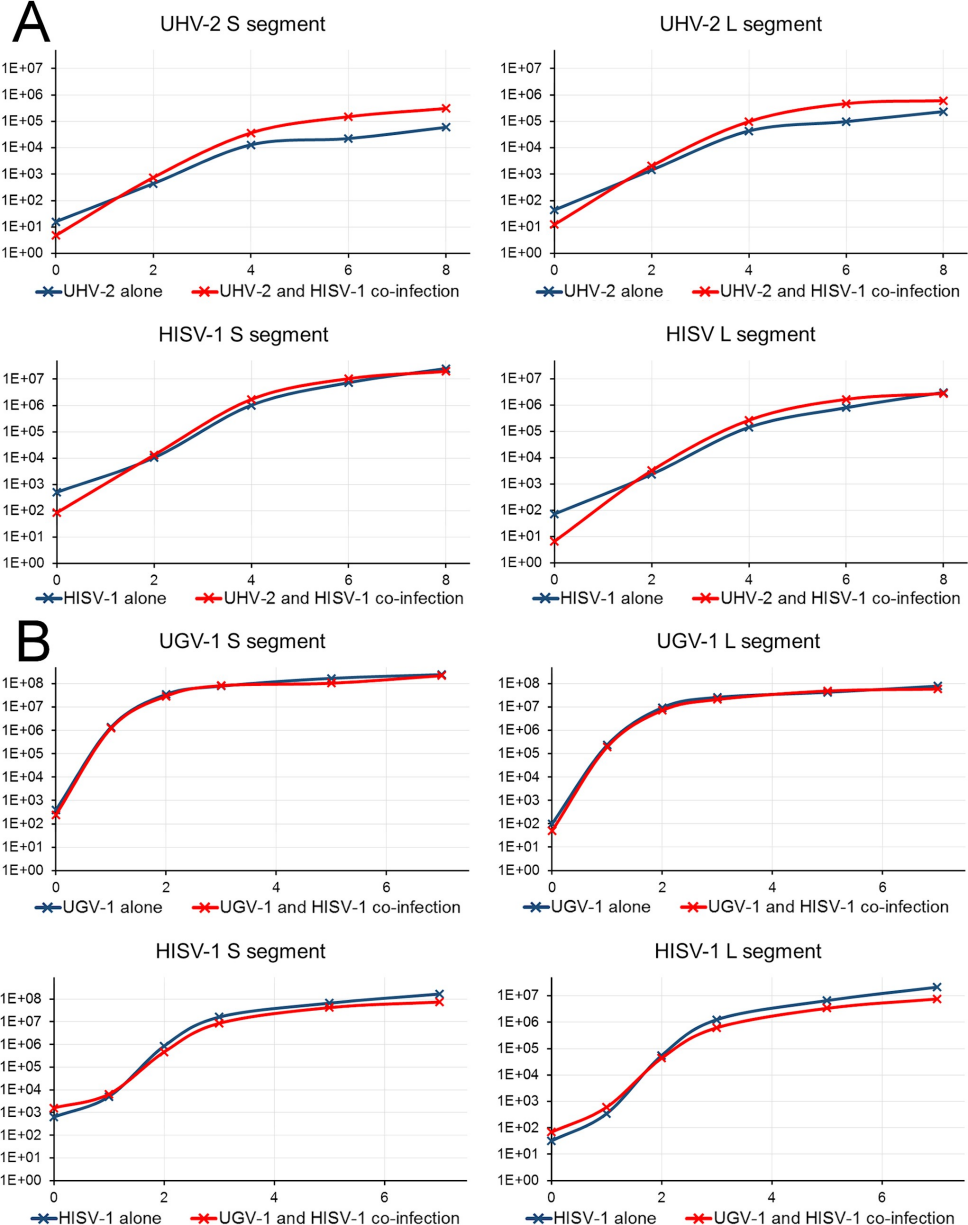
Virus production in I/1Ki cells during single virus infection vs. reptarenavirus-hartmanivirus co-infection. **A)** The supernatants of I/1Ki cells infected with UHV-2 or HISV-1 alone or co-infected with HISV-1 and UHV-2 was collected at 0, 2, 4, 6, and 8 dpi, and analyzed for the amount of viral RNA using Taqman primers and probes. The amount of S and L segment was determined individually for both viruses. The left axis shows the relative amount of virus based on delta Ct calculation. The blue curve represents the relative amount of viral RNA released in the case of UHV-2 or HISV-1 infection alone, and the red curve represents the amount of viral released RNA in co-infection. **B)** The supernatants of I/1Ki cells infected with UGV-1 or HISV-1 alone or co-infected with HISV-1 and UGV-1 was collected at 0, 1, 2, 3, 5, and 7 dpi, and analyzed for the amount of viral RNA using Taqman primers and probes. The amount of S and L segment was determined individually for both viruses. The left axis shows the relative amount of virus based on delta Ct calculation. The blue curve represents the relative amount of viral released RNA in the case of UGV-1 or HISV-1 infection alone, and the red curve represents the amount of viral RNA released in co-infection.

Since we did not see a marked difference in the amount of viral NP in western blots when comparing single-virus infection to co-infection (Fig 3A), we decided to study the effect of co-infection on virus replication by quantifying the amount of viral RNA released from infected cells, using qRT-PCR. We initially used UHV-2 and HISV-1 for the experiment, since these viruses originated from the same isolate. Fig 4A shows representative results of one of the three consecutive experiments. We found that the amount of UHV-2 RNA released in co-infection was not reduced as compared to the single virus infection (Fig 4A), indicating that co-infection does not markedly affect the replication rate of reptarenaviruses and hartmaniviruses. To provide further evidence for this observation, we performed a similar co-infection experiment using HISV-1 and UGV-1. We decided to use UGV-1, the virus of which S segment we most often find in snakes with BIBD (other authors call this S segment S6 [11]). Also, UGV-1 grows to high titers in our cell culture model. We obtained results comparable to those obtained with UHV-2 (Fig 4A and 4B), indicating that reptarenaviruses and hartmaniviruses do not interfere with each other’s replication during co-infection. We also tried infecting cultured mammalian cell lines (Baby hamster kidney, BHK-21; African green monkey kidney, Vero E6) with HISV-1 at 30°C and 37°C, however, we could not detect viral antigen or replication.

**Fig 5:**
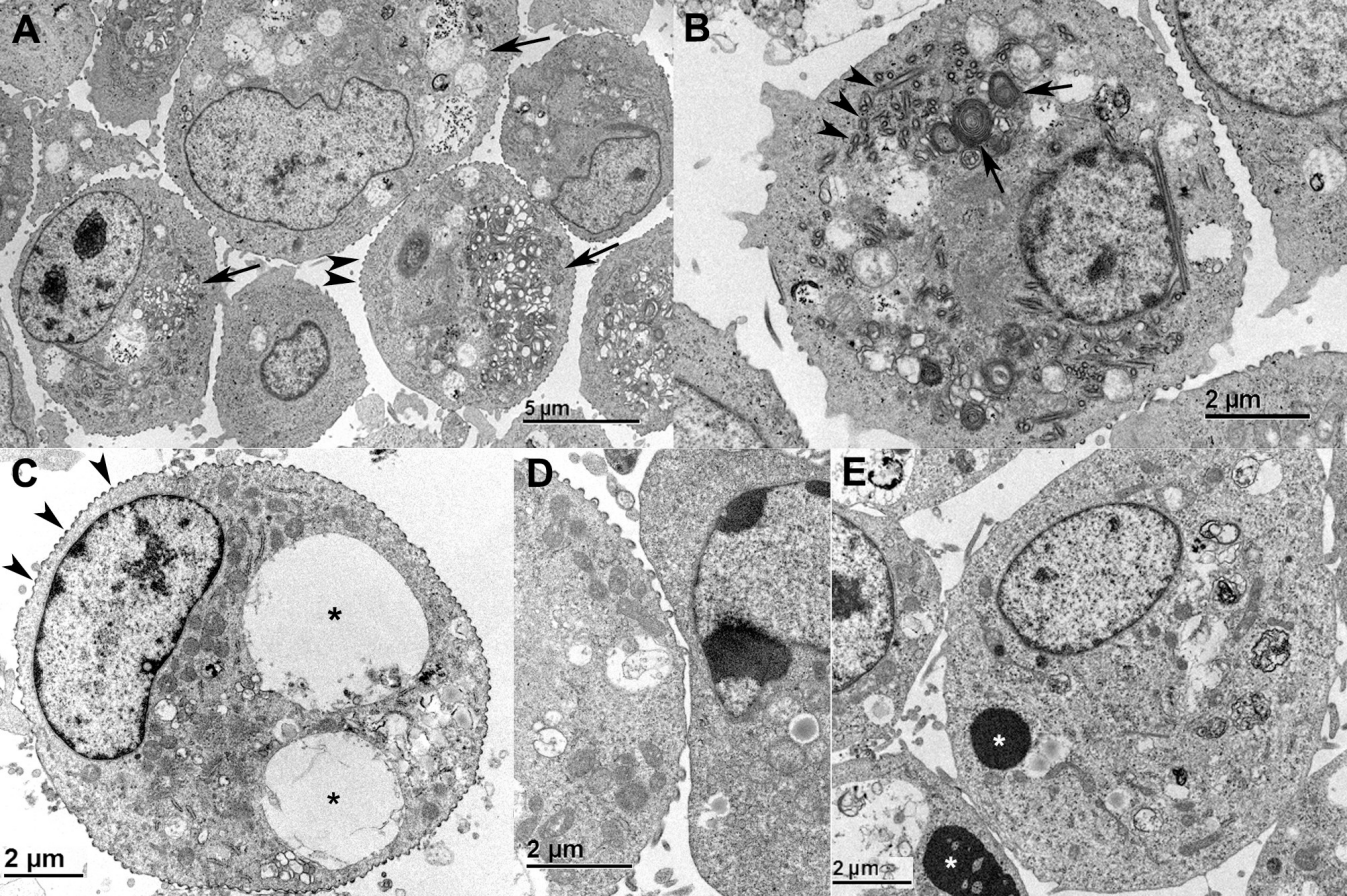
*In vitro* effects of hartmanivirus. Primary cell line derived from juvenile *B. constrictor* kidney (I/1Ki). **A-D)** HISV-1 infection, 6 dpi. **A)** Overview; cells with extensive cytoplasmic vacuolization(arrows) and multiple electron dense nodules along the cell membrane (blebbing; arrowheads). **B)** Individual cell with cytoplasmic lamellar bodies (arrows), microtubuli (arrowheads) and cytoplasmic vacuolization. **C)** Individual cells with blebbing along the cell membrane (arrowheads) and large cytoplasmic vacuoles (asterisks). **D)** Higher magnification of cell membrane blebs. **E)** UGV-1 infection, 6 dpi. Cells with characteristic cytoplasmic electron dense inclusion bodies (asterisks) and mild cytoplasmic vacuolization. There is no evidence of blebbing along the cell membrane.

**Fig 6:**
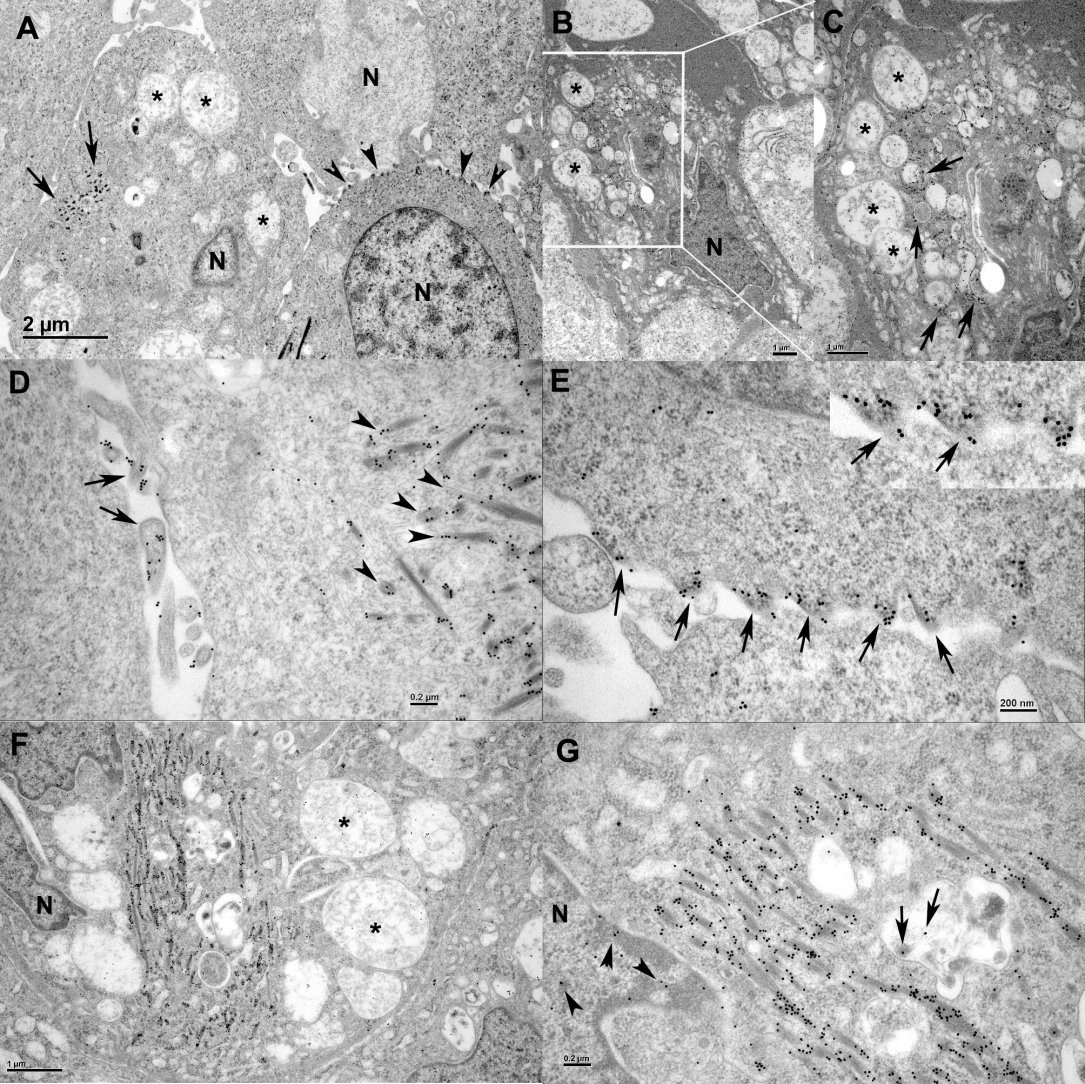
Immuno-electron microscopy (immuno-EM) of HISV-1 infected I/1Ki cells. HISV NP was visualized by anti-HISV NP antiserum and 18 nm gold-conjugated goat anti-rabbit IgG antibody. **A-C)** Cells after brief trypsin treatment prior to collection. **A)** Cells showing HISV-1 NP at cytoplasmic microtubules (arrows) and at the plasma membrane blebs (arrowheads). Cytoplasmic vacuoles are indicated by asterisks, N: nucleus. **B)** A shrunken infected cell with marked cytoplasmic vacuolization (asterisks). **C)** Higher magnification of B). The cytoplasm shows deposits of HISV-1 (arrows) adjacent to vacuoles (asterisks). **D)** A cell without trypsin treatment prior to collection. HISV-1 NP is found along cytoplasmic microtubules (arrowheads) and in association with cell protrusions (arrows). **E-G)** Cells after brief trypsin treatment prior to collection. **E)** A cell with small, partly contracted HISV-1 NP positive “blebs”at the plasma membrane (arrows). Inset: Higher magnification of the blebs, showing evidence of tubular cell-to-cell connections (arrows). **F)** A cell with abundant cytoplasmic vacuoles (asterisks) and cytoplasmic tubules containing HISV-1 NP. **G)** A higher magnification of HISV-1 NP positive tubuli in the cell presented in F. HISV-1 NP is also found within vacuoles (arrows) and around the nuclear membrane (arrowheads). N: nucleus.

**Fig 7:**
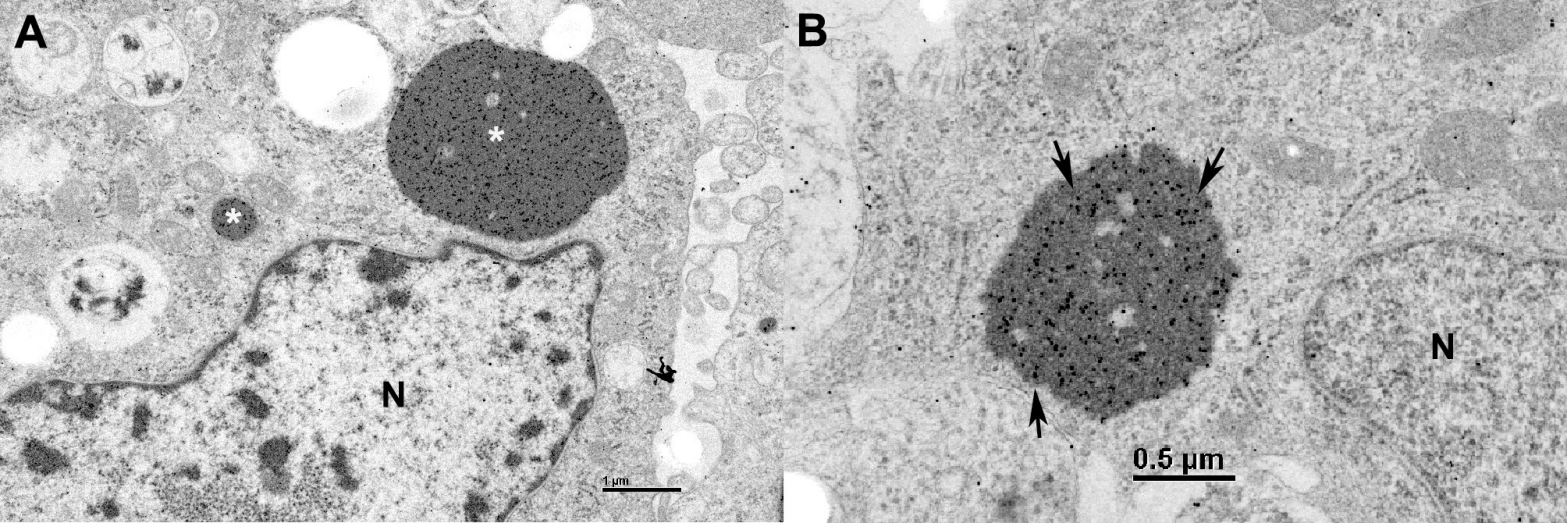
Immuno-EM of UGV-1 infected I/1Ki cells, 6 dpi. UGV-1 NP was visualized by anti-UHV NP antiserum and 18 nm gold-conjugated goat anti-rabbit IgG antibody. **A)** A cell with small and large cytoplasmic inclusion bodies containing UGV-1 NP (asterisks). **B)** A cytoplasmic inclusion body at a higher magnification demonstrating abundant UGV-1 NP (arrows).

As indicated by IF staining (Fig 3B) the reptarenavirus NP forms large IB in infected cells, which we and others have described in detail [2, 4]. However, the IF staining suggested that such IB would not be present in HISV-1 infected cells. Therefore, we performed an ultrastructural study of HISV-1 infected snake cells. Indeed, we could not demonstrate IB in HISV-1 infected cells by electron microscopy (EM). Instead, the HISV-1 infected cells generally exhibited extensive cytoplasmic vacuolization and blebbing of the plasma membrane (PM) (Fig 5A to 5C). We also observed tubular structures within the cells (Fig 5B), which appeared to contain electron electron-dense material similar to the blebs at the PM (Fig 5B to 5D). For comparison, an example of reptarenavirus infection-associated electron dense IB is presented in Fig 5E. We assumed that the electron-dense structures observed in HISV-1 infected cells would contain and/or consist of viral proteins. Indeed, immuno-EM revealed that both the tubular structures and the blebs at plasma membrane contain HISV-1 NP (Fig 6); the latter often appeared to be continuous between two adjacent cells (Fig 6E, inset) suggesting direct cell-to-cell contact. We found HISV-1 NP also associated with vacuoles (Fig 6B and 6C) and in the nucleai/along the nuclear membrane (Fig 6G), which is in line with the IF staining (Fig 3B). The HISV-1 NP was found to be abundant in the tubular structures with the infected cells (Fig 6D, 6F and 6G). Viral antigen expression in the blebs at the PM and in the intracellular tubular structures together with the detection of large foci of infected cells in IF staining could suggest that cell-to-cell spreading plays a greater role in the spread of hartmaniviruses than of reptarenaviruses. For comparison, the NP of the reptarenavirus (UGV-1) accumulated in the cytoplasmic inclusion bodies (Fig 7).

### Molecular and morphological characterization of HISV-1 virions

**Fig 8:**
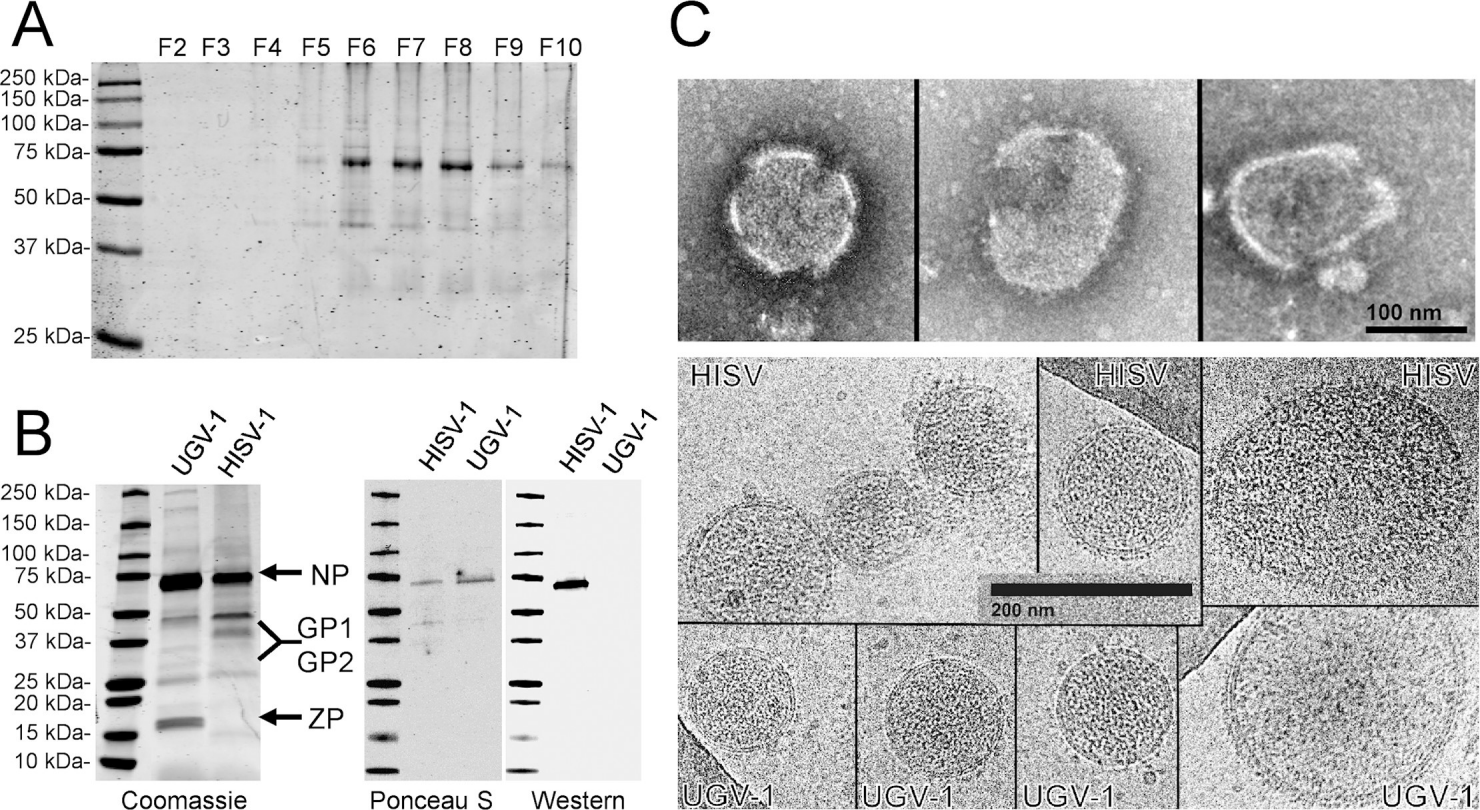
Ultrastructural characterization of HISV-1 virions. **A)** Analysis of fractions from sucrose density gradient ultracentrifugation of HISV-1 by SDS-PAGE, the proteins were visualized using Coomassie staining. The fractions were collected from the bottom of the gradient (from higher to lower density). The fractions F6-F8 contain the majority HISV-1 as judged by the intensity of ~70 kDa NP band, these fractions were pooled, concentrated and analyzed by WB (shown in panel B) to confirm the presence of NP. The molecular weight marker (Precision Plus Protein All Blue Prestained Protein Standards, Bio-Rad) is at leftmost lane. The figure was obtained using Odyssey infrared imaging system (LI-COR). **B)** The panel on right shows comparison of the proteins in purified preparation of UGV-1 (reptarenavirus) and HISV-1 (hartmanivirus), as seen by Coomassie staining. The arrows indicate the viral proteins as identified by mobility in SDS-PAGE, the label GP1 and GP2 shows the region where the processed GP1 and GP2 would likely migrate. The molecular weight marker (Precision Plus Protein All Blue Prestained Protein Standards, Bio-Rad) is at leftmost lane. The panels on right show Ponceau S (total protein) staining and WB of purified HISV-1 (pooled fractions F6-F8, panel A) and UGV-1 with anti-HISV NP antiserum. Ponceau S staining was recorded with regular flatbed scanner, the Coomassie stained gel and WB were obtained using Odyssey infrared imaging system (LI-COR). **C)** Top panel shows HISV-1 examples of virions in EM under negative staining, the scale bar is 100 nm. The bottom panel shows HISV-1 and UGV-1 as seen in cryoelectron microscopy, the scale bar is 200 nm.

Since we could not find evidence of ZP at genome level, we decided to analyze purified HISV-1 virions in comparison to those of purified reptarenavirus (UGV-1). We used UGV-1 for this approach, as it produces abundant virions in our cell culture model. For the structural study, we purified the virions by density gradient ultracentrifugation (Fig 8A). The major band in the main fractions containing HISV-1 (F6-F8 in Fig 8A) represents the NP, as demonstrated by western blotting (Fig 8B). We then compared the SDS-PAGE patterns of purified UGV-1 and HISV-1 to find more evidence on the lack of ZP in hartmaniviruses. The NP appears as the most abundant protein for both UGV-1 and HISV-1 with an approximate size of 70 kDa by SDS-PAGE mobility (Fig 8B). The exact sizes of GP1 and GP2 are not known, however, by comparing the concentrated supernatants produced by the same cell line infected with reptarenavirus vs. hartmanivirus, the indicated bands most likely represent the viral glycoproteins (Fig 8B). A doublet band at around 16–17 kDa is evident in the UGV-1 preparation, which is slightly larger than the calculated molecular weight, 12.7 kDa, of the ZP. The observed difference between predicted molecular weight (M.W.) and SDS-PAGE mobility could be due to palmitoylation, since the ZP is by prediction palmitoylated at 3 sites (http://csspalm.biocuckoo.org/online.php). As expected, the corresponding bands are missing from the HISV-1 preparation (Fig 8B), providing further evidence that hartmaniviruses lack the ZP. The result also suggests that the lack of ZP is not complemented by a similar(-sized) cellular protein. We then studied the HISV-1 virions in EM under negative staining. The virions appeared pleomorphic to roundish with an approximate diameter of 120–150 nm and most of the particles seemed not to be intact (top panels in Fig 8C). We then analyzed HISV-1 virions in cryo-EM, which provided, in agreement with the SDS-PAGE analysis, no evidence of continuous density lining the inside of the membrane (Fig 8C). A comparison of the appearance of HISV-1 and UGV-1 virions in cryo-EM confirmed that the overall morphology and size (roughly 120–150 nm in diameter) of the virions is similar (Fig 8C).

### Identification and sequencing of additional hartmanivirus species

We recently studied several *Boa constrictor* clutches born to parental animals with BIBD, aiming to demonstrate or rule out vertically transmission of reptarenaviruses [12]. We used NGS to initially define the reptarenavirome of each clutch, and confirmed our findings by species-specific RT-PCRs. However, for one of the five clutches we used only RT-PCR to demonstrate the virus transmission, since we already had analyzed three clutches from the same breeder by NGS [12]. Some of our species-specific RT-PCRs designed based on the NGS results of other clutches did not work optimally with the samples from clutch #5 [12], which prompted us to study some of the animals (snakes 2.1–2.5, Table 1) by NGS and *de novo* genome assembly. We found a hartmanivirus, designated as Veterinary Pathology Zurich virus-1 (VPZV-1), accompanied by three reptarenavirus L and two S segments in the liver of the father of this clutch (snake 2.1, Table 1) and in some of the 12 to 20-month-old offspring (snakes 2.2–2.5, Table 1) as well as one additional reptarenavirus L segment in a pooled sample of the offspring (2.2, Table 1). The GenBank accessions for the hartmaniviruses and reptarenaviruses identified in this study are provided in S1 Table. These findings suggest that also hartmaniviruses can be vertically transmitted, although we cannot entirely rule out the transmission after birth for these juvenile snakes. We also identified VPZV-1 by NGS in liver and brain samples of an adult snake from a different breeder (2.6, Table 1), again together with several reptarenavirus L and S segments. By studying some of the cell culture isolates of an earlier study [4] (animal 2.7, Table 1) with the NGS approach, and found a virus which according to ICTV criteria represents a genetically distinct lineage of the VPZV species (designated ad VPZV-2). This virus was accompanied by three reptarenavirus L and one S segments.

**Table 1:** Animals included in the study. The results of NGS and/or RT-PCR studies performed, the means of diagnosis of BIBD, age and weight, immunohistology (IH) results with anti-HISV NP antiserum, and comments/references pointing to earlier studies that included the same animal are provided.

Animal	Hartmanivirus (identified by; in)	BIBD/reptarenavirus segments	Age, weight	HISV antigen expression (IH)	Comment/references
1.1	**HISV-1** (NGS; CCS)	Pos (BS, IH) / ≥ 7 L segments (RT-PCR) in liver	Adult (6900 g)	**Brain**: disseminated individual neurons and/or axons, several ependymal cells **Peripheral nerves**: axons **Lung**: SMC, some epithelial cells **Trachea**: several epithelial cells (focal area) **Arteries** (liver, kidney, pancreas): some SMC and EC **Spleen**: neg.	**ORIGINAL HISV-1.** (plus UHV-2); Snake 11 in Table 1 and #8 in Fig 8 of [4] Case from which HISV was isolated.
1.2	**HISV-1** (RT-PCR; liver)	Questionable (BS, IH) / RT-PCR (liver) negative (UHV-like virus targeting primers)	Adult (6500 g)	**Brain**: scattered neurons and/or axons, several ependymal cells **Peripheral nerves**: axons **Lung**: SMC **Arteries** (liver, kidney, pancreas): some SMC and EC **Trachea, spleen**: neg.	Animal was housed with 1.1; snake 28 in Table 1 and #7 in Fig 8 of [4]
1.3	**HISV-1** (RT-PCR; liver)	Pos (IH) / RT-PCR positive in liver (UHV-like virus targeting primers)	Juvenile (255 g)	**Lung**: SMC (strong reaction) **Intestine**: SMC of muscle layers and arterial walls, some EC **Liver:** arteries with some SMC and EC, occasional sinusoidal EC **Brain, hear, pancreas, kidney, spleen**: neg.	Snake 10 in Table 1 and #9 in Fig 8 of [4]
1.4	**HISV-2** (NGS; brain and liver)	Pos (IH) / 3 L and 1 S segment (NGS, brain and liver)	Adult (5.5 y)	**Brain:** few neurons, ependymal cells **Lung**: SMC, epithelial cells **Trachea**: glandular epithelial cells **Stomach**: glandular epithelial cells, SMC of muscle layers **Intestine**: epithelial cells, SMC of muscle layers **Pancreas**: acinar epithelial cells **Kidney**: tubular epithelial cells **Spleen**: dendritic cells **Arteries** (liver, kidney, pancreas): some SMC and EC **Heart, thyroid, liver, testicle**: neg.	
2.1	**VPZV-1** (NGS; liver)	Pos (IH) / 3 L and 2 S segments (NGS, liver)	Adult, male	**Brain, heart, lung, stomach, intestine, liver, kidney, testicle, spleen**: neg.	**Father of clutch 5** in Table 1 of [12]
2.2	**VPZV-1** (NGS; pooled brain and liver)	Pos (BS, IH) / up to 5 L segments (RT-PCR, brain and/or liver)	Juvenile (12 mo)	NA	Offsprings of clutch 5, J5.8-J5.18 in Table 1 of [12]
2.3	**VPZV-1** (NGS; liver, in pool with 2.4)	Pos (IH) / 6 L and 1 S segment (NGS, liver, in pool with 2.4)	Subadult (18 mo)	**Neg** (brain, heart, lung, trachea, intestines, liver, pancreas, kidney, spleen)	Offspring clutch 5, J5.19 in Table 1 of [12]
2.4	**VPZV-1** (NGS; liver, in pool with 2.3)	Pos (IH) / 6 L and 1 S segment (NGS, liver, in pool with 2.3)	Subadult (18 mo)	**Neg** (brain, heart, lung, trachea, intestines, liver, pancreas, kidney)	Offspring of clutch 5, J5.20 in Table 1 of [12]
2.5	**VPZV-1** (NGS; liver)	Pos (IH) / 4 L and 2 S segments (NGS, liver)	Subadult (20 mo)	**Brain:** ependymal cells (very weak reaction) **Heart**: plexus neurons (weak reaction) **Lung, kidney:** neg.	Offspring clutch 5, J5.21 in Table 1 of [12]
2.6	**VPZV-1** (NGS; brain and liver)	Pos (IH) / 4 L and 1 S segment (NGS, brain and liver)	Adult (10 y)	**Brain**: several ependymal cells **Kidney**: tubular epithelial cells (weak reaction) **Lung**: neg.	
2.7	**VPZV-2** (NGS; CCS)	Pos (BS) / 3 L and 1 S segment (NGS, CCS)	Adult (5000 g)	NA	Snake 36 in Table 1 of [4]
3.1	**OScV-1 and OScV-2** (NGS; blood)	Pos (BS) / 5 L and 1 S segment (NGS, blood)	Adult	NA	NGS performed on RNA of pooled sera from three snakes with IBs in blood smear, Ger-pool.
3.2	**OScV-2** (NGS; blood)	Pos (BS) / 8 L and 1 S segment (NGS, blood)	Adult	NA	NGS performed on RNA of pooled sera from two snakes, F17-0012.
4.1	**DaMV-1** (NGS; brain)	Neg (BS) / 2 L and 1 S segment (NGS, brain)	Adult (>12 y)	**Lung**: SMC (weak) **Intestine**: SMC in muscle layers (weak) **Brain, trachea, thyroid, stomach, liver, pancreas, kidney, spleen**: neg.	
5.1	**Neg** (HISV-1 RT-PCR; liver)	Pos (BS, IHC) / at least 2 L segments (RT-PCR) in liver	Juvenile (170 g)	Neg (heart, lung, trachea, stomach, intestine, liver, pancreas, kidney, spleen)	Snake 9 in Table 1 and #2 in Fig 8 of [4]
5.2	**Neg** (HISV-1 RT-PCR; liver)	Pos (BS, IHC) / at least 6 L segments (RT-PCR) in liver	Juvenile (690 g)	**Neg** (brain, heart, lung, trachea, liver, intestine, kidney, spleen)	Snake 8 in Table 1 and #1 in Fig 8 of [4]
5.3	**Neg** (HISV-1 RT-PCR; liver)	Pos (BS, IHC) / at least 4 L segments (RT-PCR) in liver	Juvenile (43 g)	**Neg** (brain, lung, intestine, liver, kidney, spleen)	Snake 5 in Table 1 and #4 in Fig 8 of [4]
5.4	**Neg** (HISV-1 RT-PCR; blood)	Pos (BS, IHC) / RT-PCR positive in blood (UHV-like virus targeting primers)	Juvenile (59 g)	**Neg** (brain, lung, intestine, liver, kidney, spleen)	Snake 6 in Table 1 and #5 in Fig 8 of [4]
5.5	**Neg** (HISV-1 RT-PCR; blood)	Pos (BS, IHC) / RT-PCR positive in blood (UHV-like virus targeting primers)	Juvenile (71 g)	**Neg** (brain, lung, intestine, liver, kidney, spleen)	Snake 7 in Table 1 and #6 in Fig 8 of [4]
5.6	**Neg.** (NGS; liver)	Pos (BS, IHC) / 1 L and 1 S segment (NGS, liver)	Subadult (20 mo)	**Neg.** (brain, heart, lung, trachea, liver, kidney)	Offspring of clutch 5, J5.22 in Table 1 of [12]

BS–blood smear (used to confirm BIBD, based on the presence of cytoplasmic inclusion bodies within blood cells); IH–immunohistology; NA–not analysed; CCS–cell culture supernatant; SMC—smooth muscle cells

In another snake (1.4, Table 1) from the breeder of snake 2.6, we found a virus that by sequence comparison represents the same species as HISV-1 and thus named it HISV-2. The next hartmaniviruses we identified in a pooled blood sample of snakes (3.1, Table 1) with confirmed BIBD. According to ICTV criteria the identified viruses represent yet another hartmanivirus species, and were designated as Old Schoolhouse virus-1 and -2 (OScV-1 and -2). Subsequently, we found another representative of OScV-2 in a pooled blood sample (3.2, Table 1) of snakes with BIBD from another breeding colony. Finally, we identified a putative representative of a fourth hartmanivirus species (4.1, Table 1), which we named Dante Muikkunen virus-1 (DaMV-1), in a snake with mild neurological signs suspected to be associated with BIBD. In addition to identifying DaMV-1 and reptarenavirus segments, we also found the snake to carry a novel deltavirus, which we describe outside this report [26].

**Fig 9:**
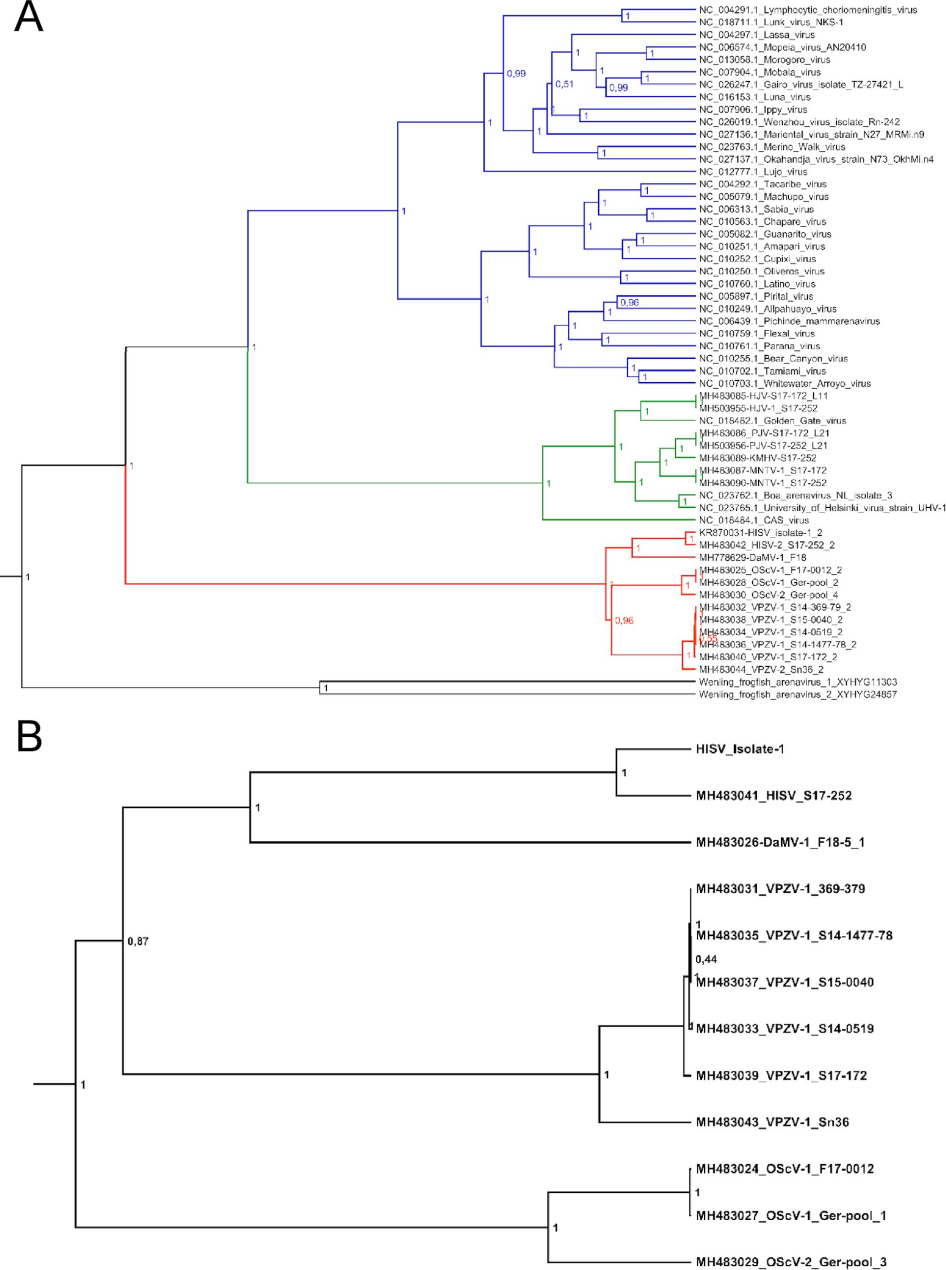
Phylogenetic analysis of hartmaniviruses. **A)** Maximum clade credibility tree of the polymerase region of arenaviruses. The tree was constructed from amino acid alignment using Bayesian MCMC method with LG model of substitution. Posterior probabilities are shown in each node. **B)** Maximum clade credibility tree of S segment of hartmaniviruses. The tree was constructed from Bayesian MCMC method with HKY model of substitution with gamma distributed rate variation among sites and proportion of invariable sites. Posterior probabilities are shown in each node.

Congruently with PASC analysis (S3 Table), the phylogenetic analyses suggested that the novel hartmaniviruses clustered according to their tentative species designations on the basis of their L and S segments (Fig 9). The phylogenetic analysis of the RdRp amino acid sequences of the representatives of all known arenavirus species suggested that the genus *Hartmanivirus* forms an outlying group separate from mammarenaviruses and reptarenaviruses, whereas recently found Wenling frogfish arenaviruses [27] form an outlying group separate from the hartmani-, reptarena- and mammarenaviruses (Fig 9).

To gain some information on the prevalence of hartmaniviruses in larger populations, we designed primers based on the L segments of OScV-1 and -2, and used RT-PCR to screen a set of 71 blood samples collected from the same breeding colony, in which we initially detected these viruses. Interestingly, 44/71 snakes were RT-PCR positive for OScV-1 and/or -2; of these, one snake was positive for both OScV-1 and -2. Thirty-four snakes in the collection were diagnosed with BIBD as they exhibited IBs in blood cells, of these, 23 had hartmanivirus infection. The fact that close to 70% of snakes with BIBD had an accompanying hartmanivirus infection indicates that the role of hartmanivirus infection in the pathogenesis of BIBD needs to be further investigated.

### Target cells of hartmanivirus in infected snakes

**Fig 10:**
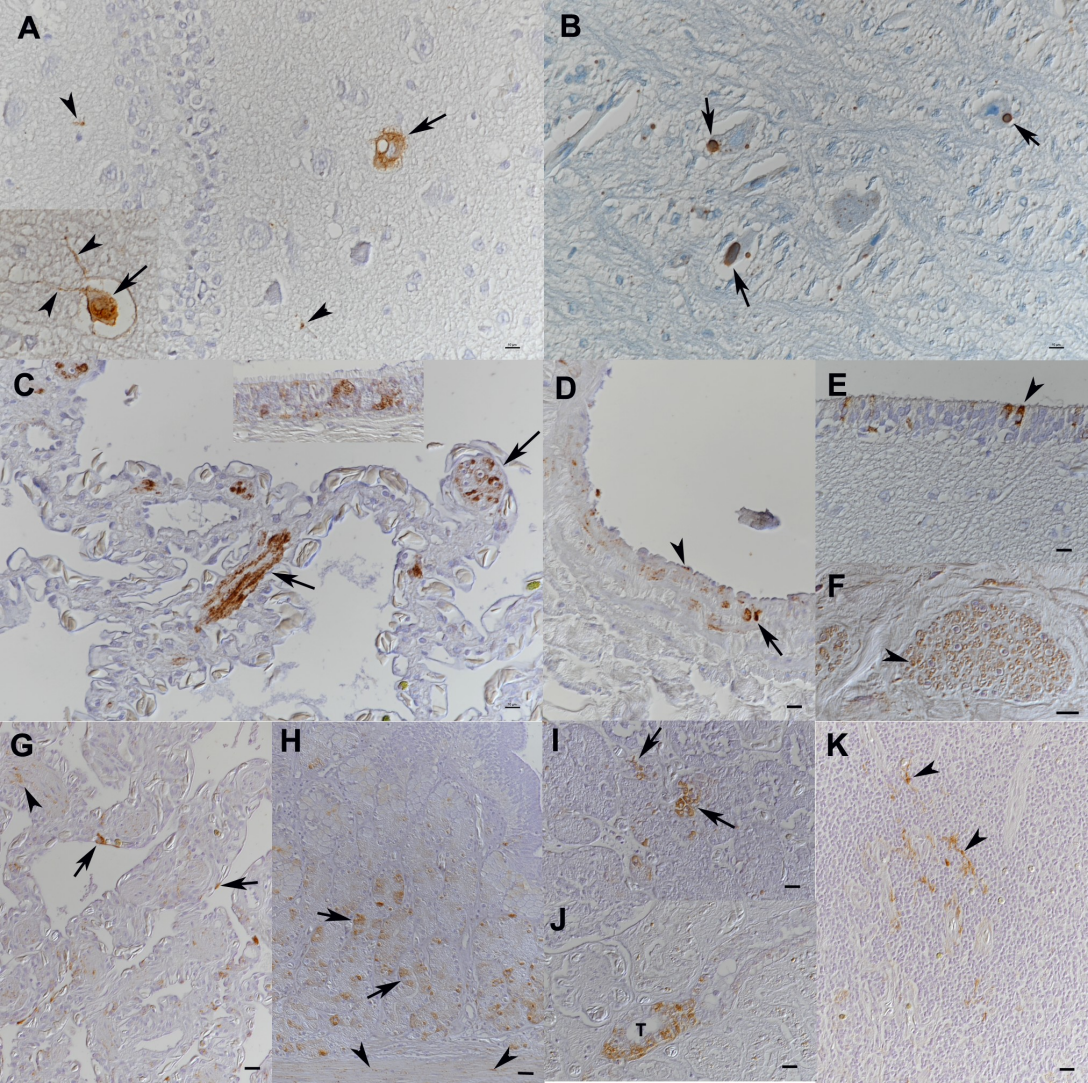
Identification of the hartmanivirus target cells *in vivo*. **A-F)** Snake 1.1. **A, B)** Brain. **A)** HISV-NP expression is seen as a granular reaction in the cytoplasm (arrows) and axons (arrowheads) of intact neurons. **B)** In contrast, expression of reptarenavirus NP is invariably seen in association with cytoplasmic, BIBD-typical inclusion bodies (arrows) in neurons. **C)** Lung. The HISV NP is abundant in smooth muscle cells (arrows). Inset: respiratory epithelium with several positive cells, exhibiting a granular cytoplasmic reaction. **D)** Large artery with smooth muscle cells positive for HISV-NP (arrow) and endothelial cell (arrow head). **E)** Brain, ependyma with several cells strongly positive for HISV-NP. **F)** Peripheral nerve, exhibiting HISV-NP expression in axons (arrowhead). **G-K)** Snake 1.4. **G)** Lung. There are HISV-NP positive epithelial cells. Arrowhead: smooth muscle cells. **H)** Stomach. The mucosa exhibits numerous glandular epithelial cells positive for HISV NP (arrows). Smooth muscle cells in the muscular layers are also occasionally positive (arrowheads). **I)** Pancreas with several HISV-NP positive acinar epithelial cells (arrows). **J)** Kidney with HISV-NP expression in epithelial cells of one tubule (T). **K)** Spleen. Several dendritic cells exhibit HISV-NP expression (arrowheads).

Production of the anti-HISV NP antibody enabled us to study the tissue and cell tropism of hartmaniviruses, using immunohistology. We initially screened the tissues of the snake from which UHV-2 and HISV-1 originate (animal 1.1, Table 1). In comparison to reptarenavirus NP, which can be detected in most tissues as cytoplasmic IB [28], HISV NP expression was very limited and most consistent in the brain. The neurons exhibited a diffuse, finely granular, cytoplasmic and/or axonal reaction (Fig 10A), which clearly differed from the reptarenavirus NP expression pattern (Fig 10B). Additionally, HISV-1 NP expression was occasionally seen in a range of other cell types: smooth muscle cells in the lung and respiratory epithelial cells (Fig 10C), endothelial cells and medial smooth muscle cells in arteries (Fig 10D), ependymal cells in the brain (Fig 10E), and axons of peripheral nerves (Fig 10F). Examination of another three snakes that harbored HISV-1 or -2 (animals 1.2–1.4) revealed a similar HISV-1 NP expression pattern, though with some variation in the range of cell types (Table 1). In animal 1.4, infected with HISV-2, a broader range of epithelial cells was found to be occasionally positive (pulmonary epithelial cells (Fig 10G), glandular epithelial cells in trachea and stomach (Fig 10H), intestinal epithelial cells, acinar epithelial cells in the exocrine pancreas (Fig 9I) and tubular epithelial cells in the kidney (Fig 10J)). Also, smooth muscle cells in the muscular layers of stomach and intestine as well as dendritic cells in the spleen were found to express the viral antigen (Figs 9 and 10). In animal 1.3, the liver exhibited HISV-1 NP expression in sinusoidal endothelial cells. In none of the hartmanivirus negative animals was there any evidence of NP expression (Table 1). We tested some of the snakes found to be infected with the above HISV-like viruses (VPZV-1: 2.1, 2.3–2.6; DaMV-1: 4.1) by IH for HISV-1 NP. The antibody appeared to cross react only minimally, as the reaction was restricted to a few individual cells in two animals (Table 1). We then tested six snakes that had been HISV-1 negative by RT-PCR (5.1–5.5) or hartmanivirus negative by NGS (5.6) for the expression of HISV-NP, all with a negative result (Table 1).

## Discussion

After a most recent revision the family *Arenaviridae* currently comprises three genera *Mammarenavirus*, *Reptarenavirus* and *Hartmanivirus* [14]. Until now the genus *Hartmanivirus* only contained HISV-1 [14], a virus that was only characterized at nucleotide level [10]. The genome of HISV-1 appeared to lack ORF for the ZP, i.e. the matrix protein of mammarenaviruses and reptarenaviruses [10]. We had isolated HISV-1 in a permanent boid kidney cell culture, however the isolate contained two viruses, UHV-2 and HISV-1. Hence, we could not confirm the lack of ZP in the initial report. It was also unclear whether HISV-1 would survive without a co-infecting reptarenavirus. Herein, we report the production of a pure HISV-1 isolate, which represents the type species of the genus *Hartmanivirus*. The successful generation of a pure HISV-1 isolate indicates that hartmaniviruses can grow in the absence of a co-infecting reptarenavirus and allowed us to study the physical properties of HISV-1 virions at nucleotide, protein and structural level. We then used HISV-1 as the model to characterize hartmanivirus infection at both *in vitro* and *in vivo* level. Furthermore, we can expand the genus *Hartmaniviruses* to four known species, by obtaining complete or near complete genome segments for three new hartmanivirus species. Finally, by screening a snake collection for two hartmaniviruses, we provide first evidence that hartmanivirus infections are rather common in captive snakes.

Comparison of the HISV-1 genome to those of viruses in the other genera of the family *Arenaviridae* shows similarities, for example in the genome ends, but also a striking difference, namely the lack of an ORF for the ZP, the matrix protein present in the other arenaviruses. We did not find additional genome segments for HISV-1 when we performed NGS on a pure preparation of HISV-1, indicating that HISV-1 comprises an S and L segment like the viruses of other arenavirus genera. We tried to seek evidence for ZP or a ZP surrogate by analyzing a pure HISV-1 preparation by SDS-PAGE and cryo-EM, but again found no evidence. The fact that we found three further hartmanivirus species (with similar coding strategy) provides further support that the lack of ZP is a general feature of the genus *Hartmanivirus*.

The ZP drives the budding of mammarenaviruses which is mediated by proline-rich late domain motifs PTAP or PPPY [19, 29]. Curiously, the ZP of the bat-borne Tacaribe virus is devoid of the late domains but does still efficiently mediate budding [30]. The ZPs of reptarenaviruses also lack the late domain, which could imply that the putative budding function of reptarenavirus ZP resides in a yet unknown motif. Interestingly, a sequence comparison reveals a conserved late motif, PPPY, in the reptarenavirus NPs, which is not found in the NPs of hartmaniviruses and mammarenaviruses. Also, the C-terminus of reptarenavirus NPs has been reported to contain late domain like motifs [2]. Thus one could speculate that also the NP associates with the budding of reptarenaviruses. Experimental studies are needed to demonstrate the role of individual proteins in the budding of reptarenaviruses, but these are beyond the scope of this report. The fact that hartmaniviruses lack a ZP, but nonetheless efficiently produce infectious particles suggests that the budding function resides in some other structural protein. Alternatively, the virus could induce a cellular protein that aids to virus budding, however we found no evidence of ZP sized proteins in purified HISV-1 preparations. Hantaviruses are similar to hartmaniviruses with regards to their proteins; they also encode RdRp, GPC and NP, but lack a matrix protein. The cytoplasmic tail of the Gn glycoprotein of hantaviruses is suggested to act as a matrix protein surrogate [31]. Thus, one could speculate that the GP2 tail of hartmaniviruses contains a motif that mediates budding. And indeed, it harbors a conserved P-Y/F-P-H-Y-P stretch (Fig 2B), which by ELM (eukaryotic linear motif resource, http://elm.eu.org/ [23]) prediction binds to the apoptosis-linked gene 2 (ALG-2) protein which bridges ALIX (ALG-2-interacting protein X) and ESCRT-I complex via interactions with ALIX, TSG101 and VPS37 [32]. Thus, hartmaniviruses might utilize the interaction with ALG-2 to gain access to the ESCRT pathway for egress, analogously to HIV-1 [33]. Our immuno-EM data suggest that the NP of hartmaniviruses is included in the buds that form along the plasma membrane of infected cells. One could thus speculate that at some point the NP of reptarenavirus ancestors obtained the budding function via emergence of late domains. This would have made the GP2 tail redundant for budding, thus explaining the lack of it in reptarenaviruses. Alternatively, it has been proposed that the GP2 of reptarenaviruses evolved from a recombination event with a filovirus or retrovirus that provided the new gene [2]. Whatever the chain of events, it seems that the ZP could have emerged around the same time to regulate replication and to bridge between the NP and the GPs to facilitate efficient genome packaging. The ZP may also have emerged before speciation of mammarenaviruses and reptarenaviruses, perhaps initially without the late domains. These hypotheses provide interesting topics for further studies, and identification of arenaviruses from other animals could help to shed light on the evolution of arenaviruses.

Comparison of the GPCs between arenavirus genera revealed that mammarenaviruses and hartmaniviruses harbor both SSP and a cytoplasmic tail in their GP2, features missing from the GPC of reptarenaviruses. The N-terminal halves of the mammarena- and hartmanivirus SSPs are more similar than the C-terminal halves. The N-terminus of the SSPs contains a myristoylation motif/site followed by a hydrophobic stretch until a conserved lysine residue in mammarenavirus or RGR motif in hartmanivirus SSPs (Fig 2B). The SSP of mammarenaviruses is suggested to span the viral membrane twice, leaving the conserved lysine residue on the virion surface [24]. Even though the C-terminal halves of hartmanivirus SSPs are less hydrophobic mammarenavirus SSPs, we hypothesize the SSPs to have a similar topology. Supporting the above, we identified a conserved cysteine residue close to the SSP C-terminus (Fig 2B), which participates in formation of an intersubunit zinc-finger structure between two conserved histidine and four cysteine residues of the GP2 [24] (Fig 2C) that are also conserved in both mammarena- and hartmaniviruses. We hypothesize these to indicate similar spike structure between mammarena- and hartmaniviruses (Fig 2C). The fact that phylogenetic analysis, as discussed below, suggests hartmaniviruses to represent the ancestors of mamm- and reptarenaviruses renders the observed differences in the GPC ORF interesting. Because the SSP and GP2 cytoplasmic tail are found in mammarena- and hartmaniviruses but not in reptarenaviruses, the reptarenaviruses seem to have lost these features at some point during their evolution, perhaps in a suggested recombination event with filo- or retroviruses [2]. The fact that GP2s of reptarenaviruses are more conserved than GP2s of mammarena- and hartmaniviruses would support both adaptation to a new niche or the speculative recombination event.

In line with the previous studies [10, 27] the phylogenetic analysis suggested that the hartmaniviruses form a basal lineage for both reptarena- and mammarenaviruses while the Wenling frogfish viruses form an outgroup to the other arenaviruses (Fig 8). Therefore, the phylogeny of arenaviruses resembles, but does not recapitulate the evolution of the respective host species. This suggests that in addition to the apparent co-evolution between arenaviruses and their hosts [27] at least one host-switch event has occurred during the evolution of arenaviruses, potentially from snakes to mammalia. Discovery of arenaviruses from other reptiles or from amphibia would shed more light on the extent of co-evolution and frequency of cross-species transmission events among arenaviruses.

Our IF studies on hartmanivirus (HISV-1), reptarenavirus (UHV-2 and UGV-1) and co-infected cell cultures showed that the distribution of NP within the infected cells varies distinctly between the two genera. We also observed that HISV-1 rapidly induced the occurrence of large foci of infected cells in our cell culture system, while UHV-2 infected cells are scattered and mostly individual. We interpreted this as evidence of more pronounced cell-to-cell spreading of HISV-1. Potential support of this interpretation is the observed bleb formation of the plasma membrane with HISV-1 (Fig 5A–5D; Fig 6A and 6E), but not with reptarenavirus (UHV-2) infection (Fig 7). The membrane blebs contained electron dense material which we assumed to be of viral origin. Indeed, immuno-EM showed that the membrane blebs contain HISV-1 NP (Fig 6E). The latter also accumulated along cytoplasmic nanoscale tubules (Fig 6D, 6F and 6G). Interestingly, influenza viruses were recently shown to utilize tunneling nanotubules (involved in intercellular communication) to transfer viral proteins and genome from infected to naive cells [34]. It is thus tempting to speculate that the NP-loaded (and likely also RNA containing) membrane blebs and cytoplasmic tubules in HISV-1 infected cells are indications that hartmaniviruses employ a similar strategy. Further studies are needed to address the above hypotheses.

The comparative investigation of snakes with BIBD by IH for reptarenavirus and HISV-1 NP provides evidence that hartmaniviruses have a more restricted cell tropism than reptarenaviruses. The mammalian kidney cells (BHK-21 and Vero E6) commonly used for propagation of mammarenaviruses were not permissive for HISV-1. However, both cell lines are permissive for reptarenaviruses when cultured at 30°C, [4, 25], which could indicate broader tissue tropism of reptarenaviruses. The frequency and extent to which hartmaniviruses were detected in neurons of infected snakes suggests a pronounced neurotropism, it therefore seems worth testing if mammalian neuronal cells would be more permissive for HISV-1. Also, further studies are needed to demonstrate the presence or absence of hartmaniviruses in snake secretions. Coincidentally, we found hartmanivirus in “clutch 5” of our previous study [12], and could demonstrate that the father and some of the juvenile offspring were carriers of the same virus. These results are indicative of vertical transmission.

We identified HISV-1 accidentally while aiming to obtain full length genomes for reptarenavirus isolates [10]. Similarly, the viruses identified herein were in the vast majority found in snakes with BIBD. Our earlier observation was that identification and full genome sequencing of reptarenaviruses works very well from brain-derived total RNA. However, in a previous study we noticed that the brain might display only a fraction of the reptarenavirus S and L segments found in the blood [12]. Due to this we have recently focused on studying liver- and/or blood-derived RNA for NGS studies, which has also led to identification of more hartmaniviruses. IH analysis of tissues from snakes with hartmanivirus infection showed that viral antigen is not abundantly expressed in the brain, which suggests that the amount of RNA in the brain is indeed low. So far we have only studied snakes with either suspected or confirmed BIBD, in which the hartmaniviruses were always accompanied by reptarenaviruses. However, by producing a pure isolate of HISV-1, we could demonstrate that hartmaniviruses do not require reptarenavirus co-infection for their infectious cycle *in vitro*. We further show that reptarena- and hartmanivirus co-infection does not negatively affect the replication of either virus. While we could not associate hartmanivirus infection with any pathological changes, further studies are needed to confirm if hartmanirviruses are apathogenic in snakes. Future studies are also needed to identify the natural host(s) of hartmaniviruses. Also, the recent discovery of a three segmented arenavirus in fish [27] indicates that more arenaviruses are yet to be found with potential to alter the understanding of arenavirus evolution.

## Materials and methods

### Ethics statement

The samples included in this study originated from animals submitted by their owners either to the Institute of Veterinary Pathology, Vetsuisse Faculty, University of Zurich, Switzerland, or to the Department of Veterinary Biosciences, Faculty of Veterinary Medicine, University of Helsinki, Finland, for a diagnostic post mortem examination. An Animals Scientific Procedures Act 1986 (ASPA) schedule 1 (appropriate methods of humane killing, http://www.legislation.gov.uk/ukpga/1986/14/schedule/1) procedure was applied to euthanize the snakes. Full diagnostic post mortem examination, blood sampling and diagnostic testing of collected samples were performed with full owners' consent. Ethical permissions for euthanasia and diagnosis-motivated necropsies (both routine veterinary procedures) were not required due to suspicion of a lethal disease, BIBD.

### Animals

The study was performed on tissues or full blood of 23 snakes that were suspected to suffer from BIBD. A further 68 blood samples from snakes in a private breeding collection which previously had animals dying with BIBD were screened for hartmanivirus infection upon the owner’s request. All animals were captive snakes from breeding collections in Germany and Switzerland, ranging in age from juvenile to more than 12 years (Table 1).

### Cells, viruses and purification of viruses

The *Boa constrictor* kidney cell line, I/1Ki, was used for virus propagation and virus isolation attempts as described [4]. A virus preparation containing HISV-1 and UHV-2 described in [10] was used as the source of pure isolates. The isolation strategy is depicted in S1 Fig. Briefly, 10-fold dilution series of the virus stock were prepared on I/1Ki cells grown on a 96-well plate, and at 14 days post infection (dpi) the cells inoculated with virus dilutions 1:10^7^ and 1:10^8^ were transferred onto a 24-well plate. The cell culture medium was collected at 7 and 14 dpi, and the pooled cell culture supernatants were analyzed by virus species specific RT-PCR as described [10, 12]. For production of HISV-1 stock, a 75-cm^2^ flask of semi-confluent I/1Ki was inoculated with 500 μl of the pooled supernatant from the 24-well plate, the cell culture medium was collected and replaced at 2–3 day intervals until 14 dpi, and the pooled supernatants were filtered through a 0.45 μm syringe filter (Millipore) and stored at -80°C for further use. Large quantities of HISV-1 were produced by inoculating semi-confluent I/1Ki 75-cm^2^ flasks with 1 ml of 1/50-1/200 diluted HISV-1 stock, followed by supernatant collection as described above. Viruses were concentrated by pelleting through a sucrose cushion and more pure virus preparations were obtained by sucrose density gradient ultracentrifugation as described [4, 35, 36]. For cryo-electron microscopy (cryo-EM) the virus-containing fractions were pooled and dialyzed against phosphate-buffered saline (PBS). For transmission electron microscopy and immunocytology, cells were infected and harvested six days after inoculation, pellets prepared and fixed as described [4]. For co-infection experiments I/1Ki cells were inoculated either with equal amounts of HISV-1 and UHV-2, or HISV-1 and UHV-2 alone as controls (multiplicity of infection > 1). The infection vs. co-infection experiments were done in duplicate.

### Isolation of RNA

Trizol and Trizol LS isolation reagent (Life Technologies) in combination with QiaGEN RNeasy Mini Kit (Qiagen) was used for RNA isolation as described [12]. RNA isolation from cell culture supernatants was done with either the QIAamp Viral RNA Mini Kit (Qiagen) or the GeneJET RNA Purification Kit (Thermo Fisher Scientific) following the manufacturer’s instructions. No carrier RNA was used during RNA isolation for samples analyzed by NGS. RT-PCR served to detect viral RNA from cell culture supernatants as described [10, 12].

### Circularization of RNA, reverse transcription-polymerase chain reaction (RT-PCR), and sequencing of the genome ends

HISV-1 RNA isolated from pelleted virus was treated with T4 polynucleotide kinase (Thermo Fisher Scientific) according to the manufacturer’s protocol, purified using the QiaGEN RNeasy Mini Kit (Qiagen), and circularized with T4 RNA ligase (Thermo Fisher Scientific). The RNA circularization reaction (2 h at 25°C) mix (20 μl in DEPC-treated water) included: 2 μl of 10X reaction buffer, 5 μl of isolated RNA, 1 μl of T4 RNA ligase, 0.5 μl RNAse inhibitor (40 μ/μl), 10% PEG 8000, and 100 μM ATP. The reaction ligase was inactivated by heating the reaction mix to 70°C for 10 min. The circularized RNA was reverse transcribed using RevertAid H Minus Reverse Transcriptase (Thermo Fisher Scientific) following the manufacturer’s protocol for specific primers. The S segment primers were 5´-CTCCATTTACTCGAACAAGCTCAC-3´ and 5´-CAGGTTAAATTCATTGTTGGAGCA-3´, the L segment primers were 5´-GCACAACAATCTTTCTGCGAT-3´ and 5´-CAGGGCTTTGTTTTGTCCAG-3´. Phusion Flash High-Fidelity PCR Master Mix (Thermo Fisher Scientific) was used for PCR amplification. The reaction mix consisted of: 1 μl of cDNA, 10 μl of Master Mix, 1 μl of forward and reverse primer (10 μM stocks), and 7 μl of molecular grade water. The cycling conditions were: 1) 10 s at 98°C; 2) 1 s at 98°C; 3) 5 s at 60°C; 4) 7 s at 72°C; 5) 1 min at 72°C; steps 2 to 4 were repeated 35 times. The PCR products were separated by agarose gel electrophoresis, purified with the QIAquick gel extraction kit (Qiagen) following the manufacturer’s instructions, and cloned into plasmid using Zero Blunt TOPO PCR Cloning Kit (Thermo Fisher Scientific) following the manufacturer’s recommendations. Plasmid minipreps were purified using the GeneJET Plasmid Miniprep Kit (Thermo Fisher Scientific) and the purified plasmids were sent for sequencing (with M13 forward and reverse primers) to Microsynth (Zurich, Switzerland).

### *In silico* structure prediction for genome ends

To study the secondary structures formed by the genome ends of LCMV (strain Armstrong 53b, S segment GenBank accession NC_004294, L segment NC_004291), GGV (S segment NC_018483, L segment NC_018482), and HISV-1 (S segment KR870017, L segment KR870031) we used DuplexFold Web Server of RNA structure at RNAstructure (Web Servers for RNA Secondary Structure Prediction, available at https://rna.urmc.rochester.edu/RNAstructureWeb/Servers/DuplexFold/DuplexFold.html) [37, 38]. We applied standard parametrization for the predictions, the folding free energies for the models chosen for presentation are: LCMV S segment -30.9 kcal/mol; LCMV L segment, -41.4 kcal/mol; GGV S segment -33.4 kcal/mol; GGV L segment -35.6 kcal/mol; HISV S segment -34.3 kcal/mol; and HISV L segment, -29.9 kcal/mol.

### Next-generation sequencing (NGS)

NGS and *de novo* assembly was done as described [12, 39].

### Phylogenetic analysis

The sequences were aligned with Clustal Omega algorithm [40] implemented in EMBL-EPI webserver [41]. The phylogenetic trees were constructed using Bayesian Monte Carlo Markov Chain (MCMC) method implemented in BEAST version 2.4.7 [42] using LG or HKY-G-I substitution models for amino acid and nucleotide sequences, respectively. The analyses were run for 50 million states and sampled every 5000 steps. They were carried out on the CSC server (IT Center for Science Ltd., Espoo, Finland). Posterior probabilities were calculated with a burn-in of 10% and checked for convergence using the Tracer version 1.6.

### Production of recombinant HISV NP, purification, and production of antiserum

Full-length NP (amino acids 1–582), and N- (aa 1–295) and C-terminal (aa 296–582) parts of it were PCR cloned for *E*. *coli* expression using primers (forward for full length and N-terminal portion 5´-CACCATGTCCTTGAACAAGGACCTT-3´; reverse for N-terminal portion 5´- TCTGTCGCTGGTGCAACC-3´; forward for C-terminal portion 5´- CACCATGATCTCATCTCAAAACATACC-3´; reverse for full length and C-terminal portion 5´- GTTGTTCATTATGTAGTTGAA-3´) designed according to the Champion pET101 Directional TOPO Expression Kit with BL21 Star (DE3) One Shot Chemically Competent *E*. *coli* manual (Thermo Fisher Scientific). Protein production and purification was done as described [39]. Full length HISV NP could not be recovered by this strategy, but the N- and C-terminal portions were recovered in moderate and good amount, respectively. The purified C-terminal portion of HISV NP was dialyzed against PBS. A rabbit polyclonal antiserum against C-terminal portion HISV NP (anti-HISV NP-C) was produced by Biogenes GmbH (Berlin, Germany).

### Immunofluorescence (IF) staining

IF staining was done on cells grown on 24-well Glass Bottom Plates (In Vitro Scientific) as described [39]. The primary antibodies, anti-UHV NP-C or anti-HISV NP-C antisera, were used at 1:2,000 dilution in PBS.

### SDS-PAGE and western blotting

Routine protocols, described in [43], were utilized for SDS-PAGE and western blotting, the results were recorded with the Odyssey Infrared Scanning System (LI-COR).

### Quantitative reverse transcription-polymerase chain reaction (qRT-PCR)

A Taqman qRT-PCR assay for quantifying the S and L segments of UHV-2 and HISV-1 served to monitor the growth of UHV-2 and HISV-1 in cell culture. The primers and probes were: UHV-2 S segment forward primer (FWD) 5´-GCAAAACAGAACTGCTGCAGTC-3´, reverse primer (REV) 5´-TGCGATACAGACATAATTAGAGACATTG-3´, and probe 5´-6-Fam(carboxyfluorescein)-GTCACCATGTGTCCCTCAGAACTCATTCA-3´-BHQ-1 (Black Hole Quencher); UHV-2 L segment FWD 5´-TTGGGGAGTTTGTTACCAATGT-3´, REV 5´- GTGGGCCCAAATAACAAACCT-3´, and probe 5´-6-Fam- CTCTCTCGGACCTCCCACTTGTTCCTTTATG-3´-BHQ-1; UGV-1 S segment forward primer (FWD) 5´- CAAGAAAAACCACACTGCACA-3´, reverse primer (REV) 5´- AACCTGTTGTGTTCAGTAGT-3´, and probe 5´-6-Fam(carboxyfluorescein)- CTCGACAAGCGTGGGCGGAGG-3´-BHQ-1 (Black Hole Quencher); UGV-1 L segment FWD 5´- TCATAAAAGCTTCTAGCTATTCTTTTCAT-3´, REV 5´- CAAGTTGGAGGCCCAAGAG-3´, and probe 5´-6-Fam- TGAAGTCTCCTCCAAGACCCTGGTTATCAG-3´-BHQ-1; HISV-1 S segment FWD 5´-CTCAAAATCTTACCGAAGTTGTATGTAC-3´, REV 5´-CACTTTCCCTTTTGGATCTTTG-3´, and probe 5´-6-Fam-GTGACGACCAAGTGTCGGGTCACAC-3´-BHQ-1; HISV-1 L segment FWD 5´-GAGTCTTTGTTTGATAATGGTGGTT-3´, REV 5´-ATTGAAGACTACAGAACCATATC-3´, and probe 5´-6-Fam-TCATTTGATTCAAGTGTTCTCAGGGCA-3´-BHQ-1 (Metabion International Ag). RNA isolation for Taqman assays was done with the GeneJET RNA purification kit (Thermo scientific) with carrier RNA following the manufacturer’s protocol. Taqman Fast Virus 1-step master mix (Thermo scientific) was used for qRT-PCR, 10 μl reactions were run with the AriaMX real-time PCR System (Agilent) in duplicate with recommended cycling conditions: 1) 50°C 5 min; 2) 95°C 20 s; 3) 95°C 3 s; 4) 60°C 30 s (steps 3 and 4 were repeated 39 times).

### Transmission electron microscopy (EM) and immune-EM

Pellets from HISV-1 or UGV-1 infected I/1Ki cells were fixed in 1.5% glutaraldehyde, buffered in 0.2 M cacodylic acid buffer, pH 7.3 for 12 h at 5°C and embedded in epoxy resin. Toluidin blue stained semithin (1.5 μm) sections and, subsequently, ultrathin (100 nm) sections were prepared and the latter contrasted with lead citrate and uranyl acetate and examined with a Philips CM10 transmission electron microscope at 80kV.

For immuno-EM, cell pellets were fixed in 2.5% glutaraldehyde in 0.5xPBS and epoxy resin embedded. Thin sections (100 nm) were prepared and incubated for 30 min at RT in PBS with 1% BSA, followed by overnight incubation with rabbit anti-HISV NP antiserum (diluted 1:1,000 in PBS with 1% BSA) at 4°C. After washing with PBS, sections were incubated with 18 nm gold-conjugated goat anti-rabbit IgG antibody (Milan Analytica AG, Rheinfelden, Switzerland; diluted 1:20 in PBS with 1% BSA) for 2 h at RT. Sections were then contrasted and examined as descrived above.

### Histology and immunohistology

Immunohistology with anti-HISV NP antiserum at 1:6,000 dilution was performed on formalin-fixed, paraffin embedded (FFPE) tissue sections, following previously published protocols [4, 12, 39]. Refer to Table 1 for animals examined. Consecutive sections incubated with a non-reactive rabbit polyclonal antibody instead of the specific primary antibody served as negative controls. A further section of each block was stained for reptarenavirus NP as described [4]. A FFPE pellet prepared from each an HISV- and reptarenavirus-infected cell sample served as positive control for the immunohistological stains. All snakes were also examined for any histopathological changes, using hematoxylin-eosin stained consecutive sections.

### Accession numbers

The virus sequences obtained in this study are made available via GenBank, the accession numbers are provided in S1 Table.

## Supporting information

S1 Fig Schematic flow of the strategy used to obtain clean UHV-2 and HISV-1 isolates. I/1Ki cells grown on 96-well plate were overlayed with 10-fold dilution of virus stock containing both viruses. At 14 dpi the cells on rows F and G were transferred onto 24-well plate. The supernatants collected at 7 and 14 dpi were pooled, and analyzed by RT-PCR with primers specific to UHV-2 and HISV-1. The results for the lowest dilution are shown at the bottom, the circled bands represent pure the pure isolates of HISV-1 and UHV-2.
(PDF)

S2 Fig Schematic illustration of the sequencing strategy for HISV-1 genome ends. The viral RNA isolated from cell culture supernatant was treated with polynucleotide kinase (PNK) to ascertain the phosphorylation of 5´ end before treatment with T4 RNA ligase I. The ligated RNA was transcribed to cDNA using primer specific for either S or L segment, after which the genome ends were PCR amplified. The PCR products were cloned into a plasmid, and individual clones were Sanger sequenced. The coverage at which HISV-1 L and S segments were sequenced by NGS are shown below. The coverage was obtained by aligning reads to full length S and L segment of HISV-1 by using Bowtie2 in Unipro UGENE v.1.25.0.
(PDF)

S3 Fig Coverages of hartmaniviruses identified. **A)** Coverage of HISV-1 (pure isolate) and HISV-2, snake 1.4 (Table 1). **B)** Coverages of VPZV-1 L and S segments, snakes 2.1, 2.2, 2.3, 2.5, 2.6 and 2.7 (Table 1). **C)** Coverage of OScV-1 and OScV-2 L and S segments, snakes 3.1 and 3.2 (Table 1). **D)** Coverage of DaMV-1 S segment, snake 4.1 (Table 1).
(PDF)

S4 Fig Coverages of reptarenaviruses identified. (PDF)

S1 Table Names, abbreviations, and accession numbers for viruses sequenced in this study. (PDF)

S2 Table Proteomes and amino acid identities between type species of family *Arenaviridae* genera. (PDF)

S3 Table Results of PASC analyses with HISV-1 S and L segments alone, and with all hartmanivirus L and S segments included. (PDF)
