# Supplementary material for: Characterization of Haartman Institute snake virus-1 (HISV-1) and HISV-like viruses—The representatives of genus Hartmanivirus, family Arenaviridae
Source: PLoS Pathog. 2018 Nov 14;14(11):e1007415. doi: 10.1371/journal.ppat.1007415 (PMC6261641; doi:10.1371/journal.ppat.1007415)
Supplement: S1 Fig — I/1Ki cells grown on 96-well plate were overlayed with 10-fold dilution of virus stock containing both viruses. At 14 dpi the cells on rows F and G were transferred onto 24-well plate. The supernatants collected at 7 and 14 dpi were pooled, and analyzed by RT-PCR with primers specific to UHV-2 and HISV-1. The results for the lowest dilution are shown at the bottom, the circled bands represent pure the pure isolates of HISV-1 and UHV-2. (PDF) [file ppat.1007415.s001.pdf]

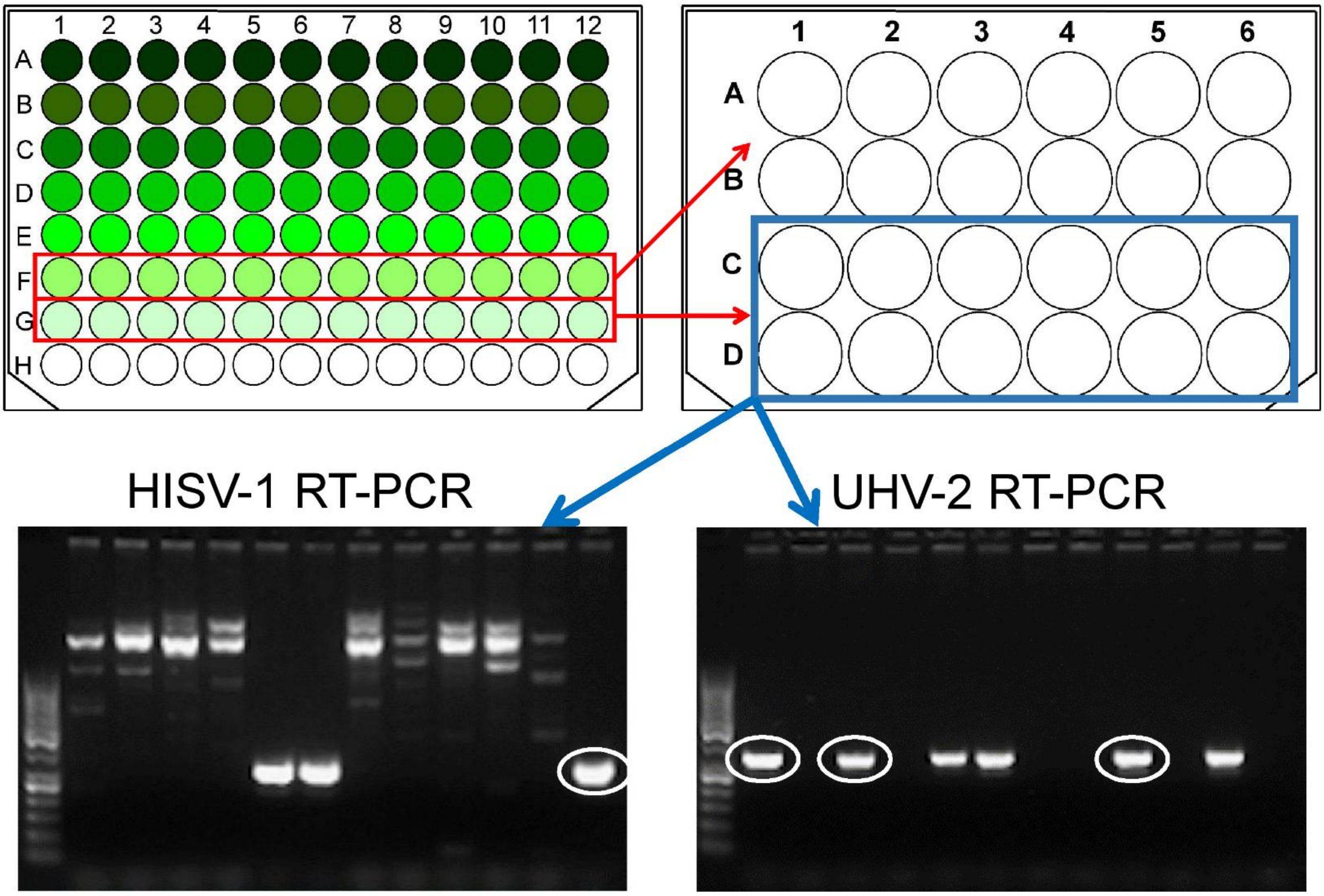

Supplementary figure 1. Schematic flow of the strategy used to obtain clean UHV-2 and HISV-1 isolates. I/1Ki cells grown on 96-well plate were overlayed with 10-fold dilution of virus stock containing both viruses. At 14 dpi the cells on rows F and G were transferred onto 24-well plate. The supernatants collected at 7 and 14 dpi were pooled, and analyzed by RT-PCR with primers specific to UHV-2 and HISV-1. The results for the lowest dilution are shown at the bottom, the circled bands represent pure the pure isolates of HISV-1 and UHV-2.
