# Supplementary material for: Characterization of Haartman Institute snake virus-1 (HISV-1) and HISV-like viruses—The representatives of genus Hartmanivirus, family Arenaviridae
Source: PLoS Pathog. 2018 Nov 14;14(11):e1007415. doi: 10.1371/journal.ppat.1007415 (PMC6261641; doi:10.1371/journal.ppat.1007415)
Supplement: S2 Fig — The viral RNA isolated from cell culture supernatant was treated with polynucleotide kinase (PNK) to ascertain the phosphorylation of 5´ end before treatment with T4 RNA ligase I. The ligated RNA was transcribed to cDNA using primer specific for either S or L segment, after which the genome ends were PCR amplified. The PCR products were cloned into a plasmid, and individual clones were Sanger sequenced. The coverage at which HISV-1 L and S segments were sequenced by NGS are shown below. The coverage was obtained by aligning reads to full length S and L segment of HISV-1 by using Bowtie2 in Unipro UGENE v.1.25.0. (PDF) [file ppat.1007415.s002.pdf]

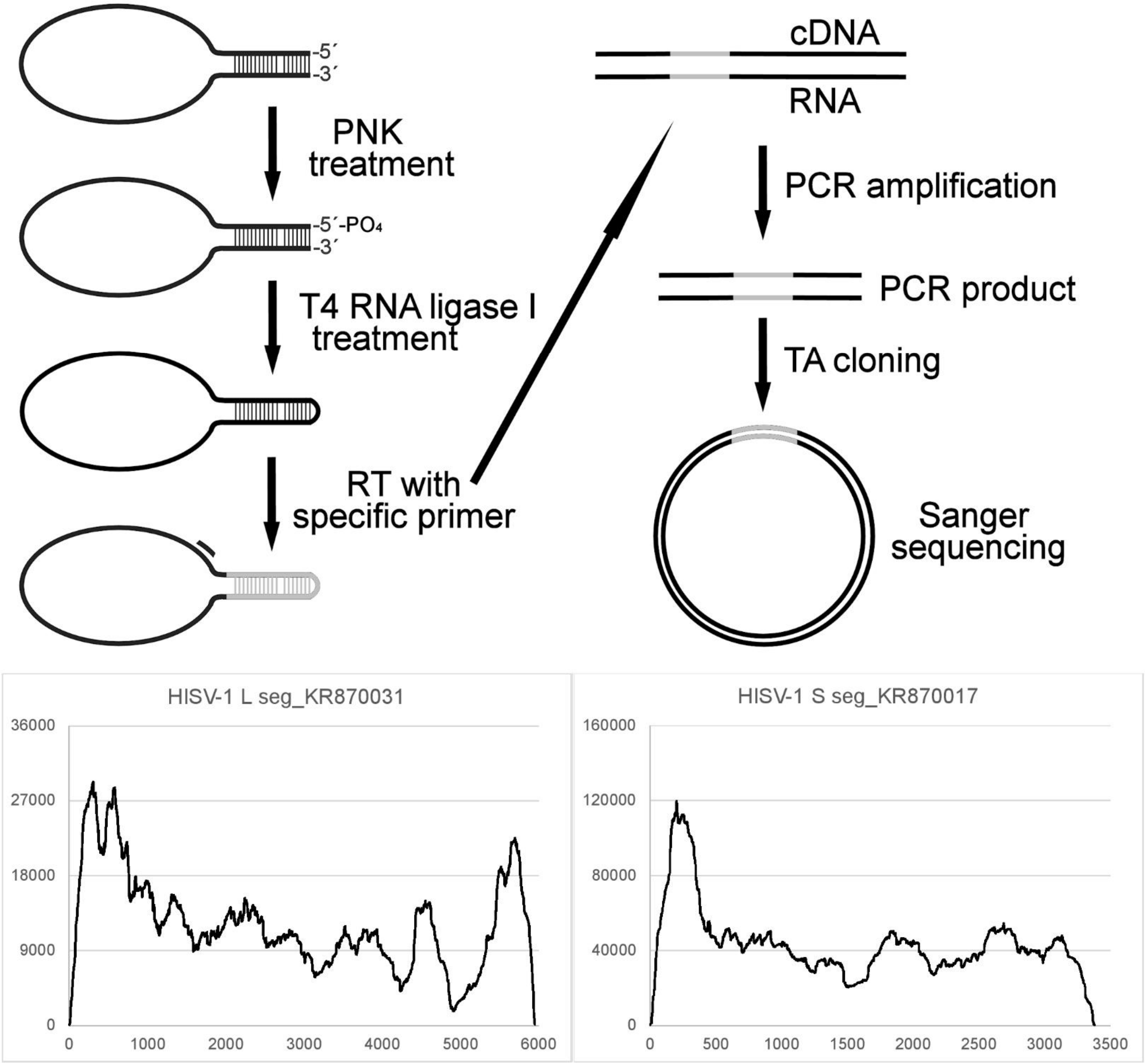

Supplementary figure 2. Schematic illustration of the sequencing strategy for HISV-1 genome ends. The viral RNA isolated from cell culture supernatant was treated with polynucleotide kinase (PNK) to ascertain the phosphorylation of 5´ end before treatment with T4 RNA ligase I. The ligated RNA was transcribed to cDNA using primer specific for either S or L segment, after which the genome ends were PCR amplified. The PCR products were cloned into a plasmid, and individual clones were Sanger sequenced. The coverage at which HISV-1 L and S segments were sequenced by NGS are shown below. The coverage was obtained by aligning reads to full length S and L segment of HISV-1 by using Bowtie2 in Unipro UGENE v.1.25.0.
