# Supplementary material for: Characterization of Haartman Institute snake virus-1 (HISV-1) and HISV-like viruses—The representatives of genus Hartmanivirus, family Arenaviridae
Source: PLoS Pathog. 2018 Nov 14;14(11):e1007415. doi: 10.1371/journal.ppat.1007415 (PMC6261641; doi:10.1371/journal.ppat.1007415)

A) Coverage of HISV-1 (pure isolate) and HISV-2, snake 1.4 (table 1).

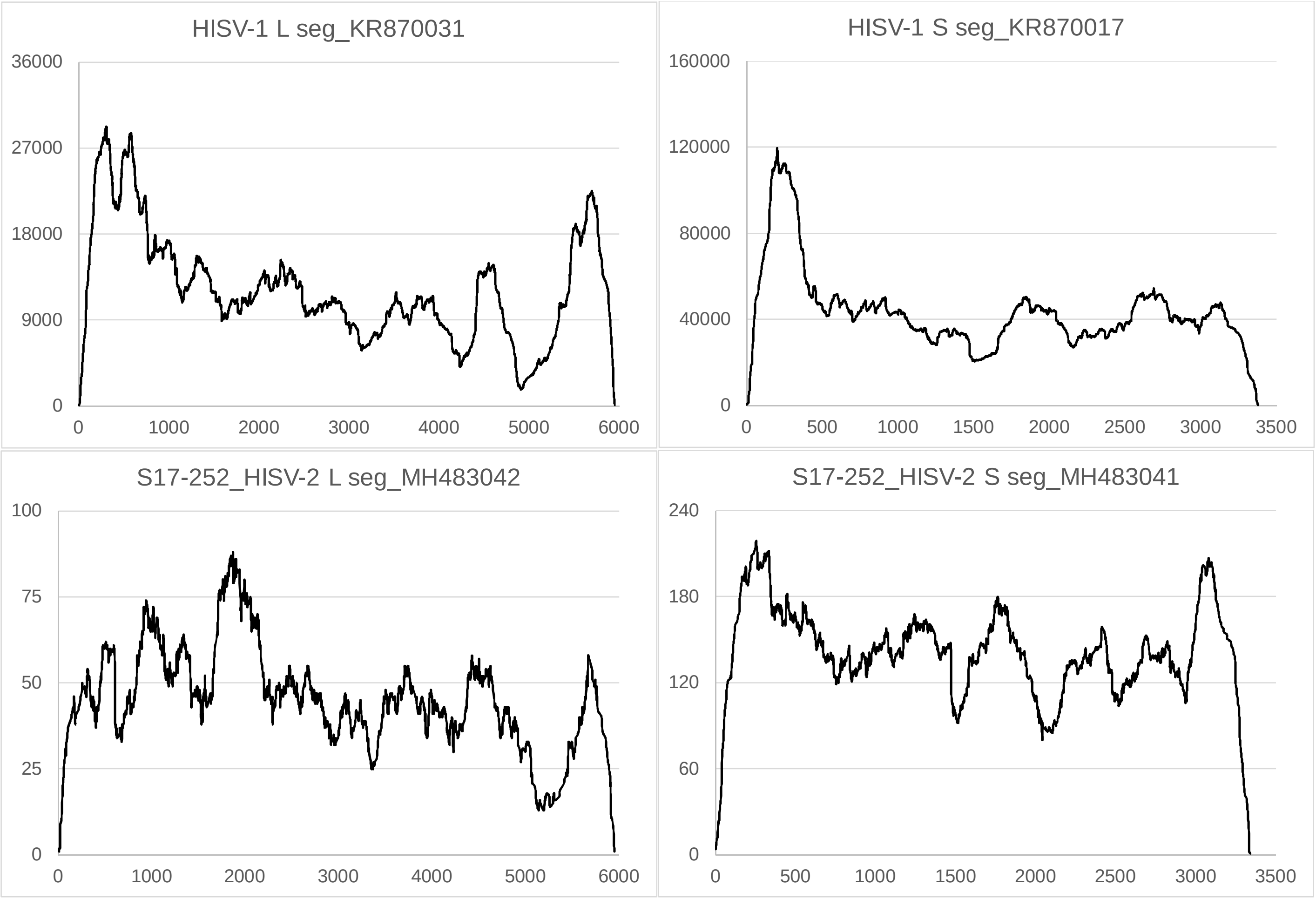

B) Coverages of VPZV-1 L and S segments, snakes 2.1, 2.2, 2.3, 2.5, 2.6 and 2.7 (table 1).

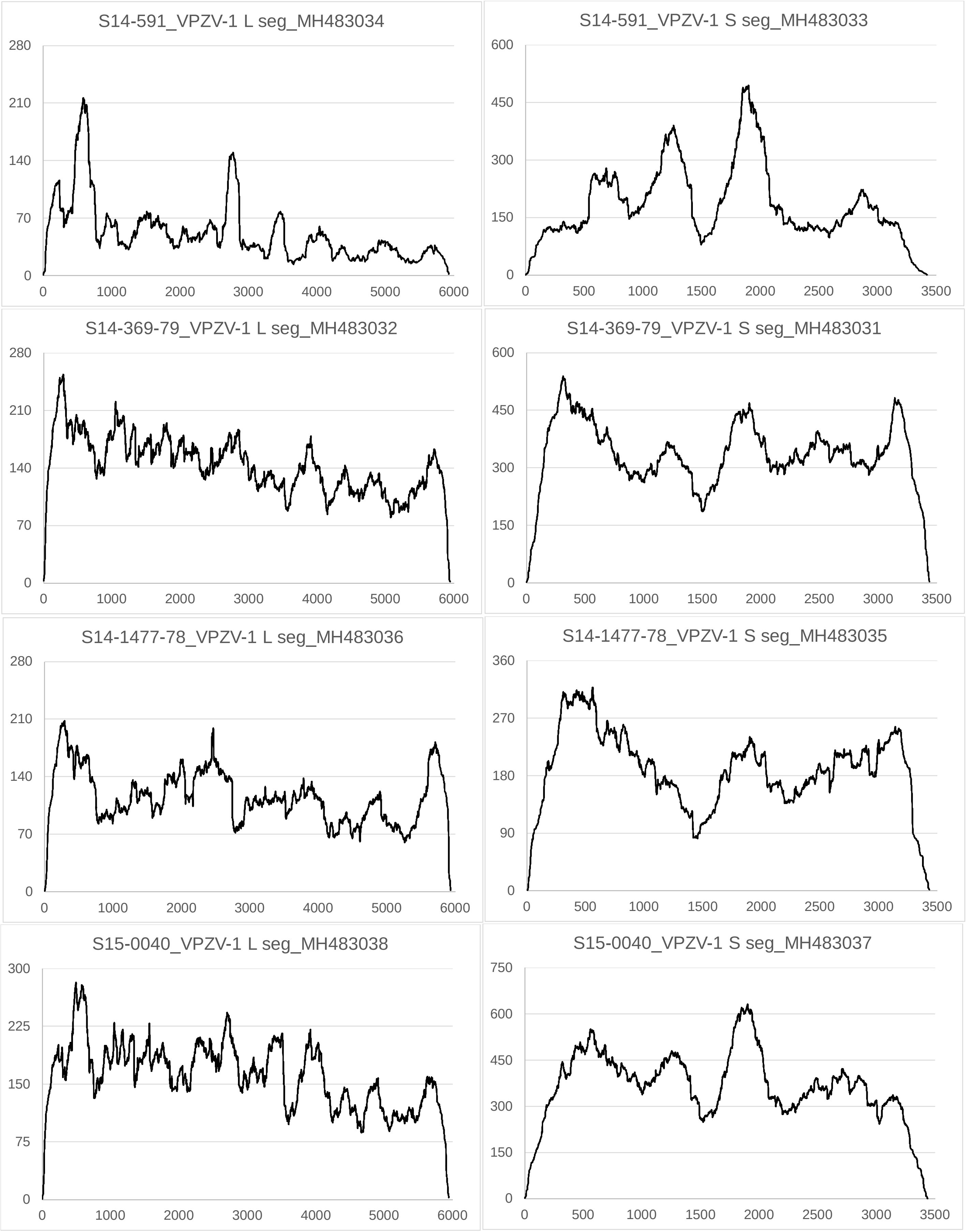

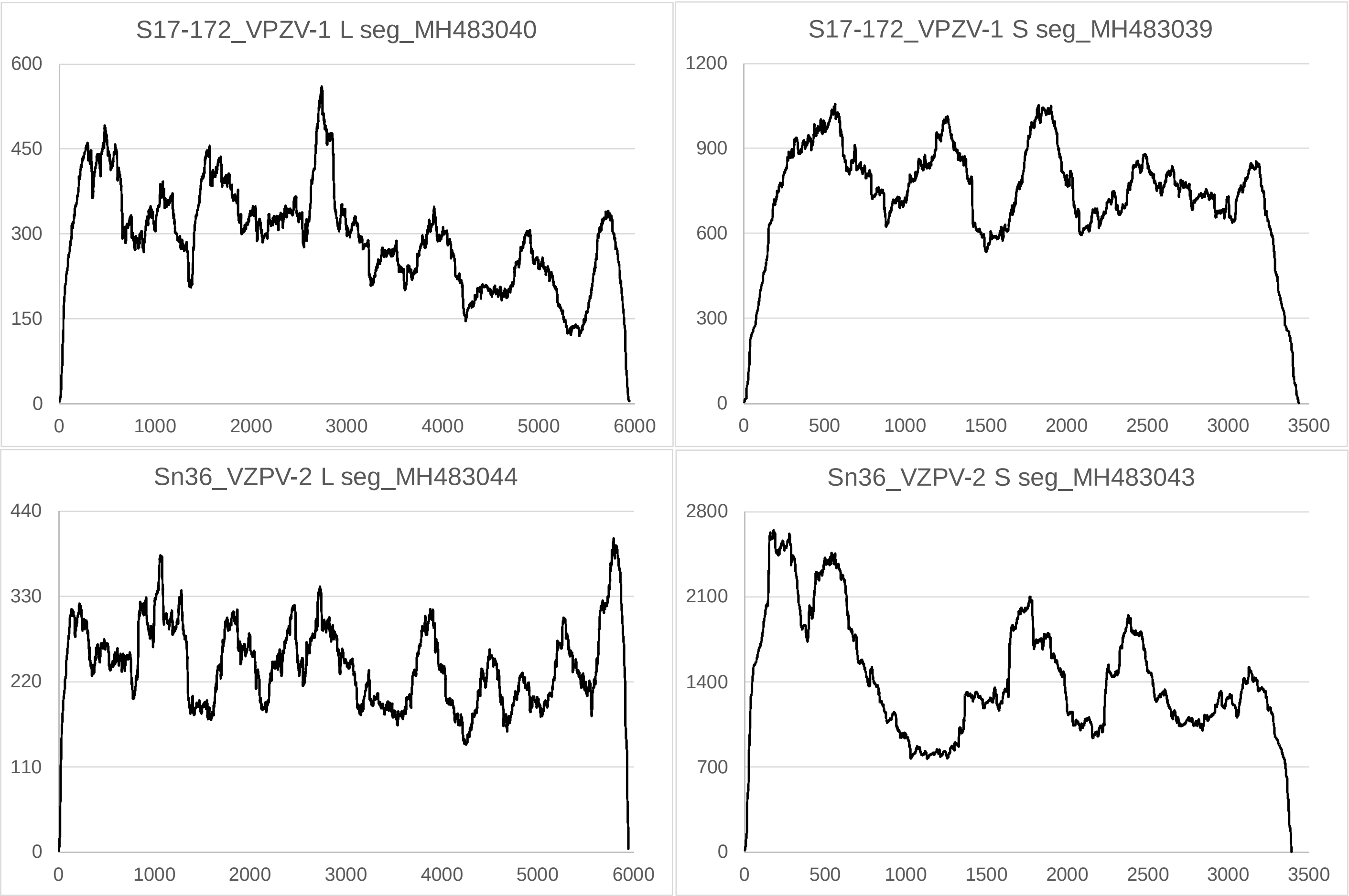
S17-172_VPZV-1 L seg_MH483040
0
150
300
450
600
0
1000
2000
3000
4000
5000
6000
S17-172_VPZV-1 S seg_MH483039
0
300
600
900
1200
0
500
1000
1500
2000
2500
3000
3500
Sn36_VZPV-2 L seg_MH483044
0
110
220
330
440
0
1000
2000
3000
4000
5000
6000
Sn36_VZPV-2 S seg_MH483043
0
700
1400
2100
2800
0
500
1000
1500
2000
2500
3000
3500

C) Coverage of OScV-1 and OScV-2 L and S segments, snakes 3.1 and 3.2 (table 1).

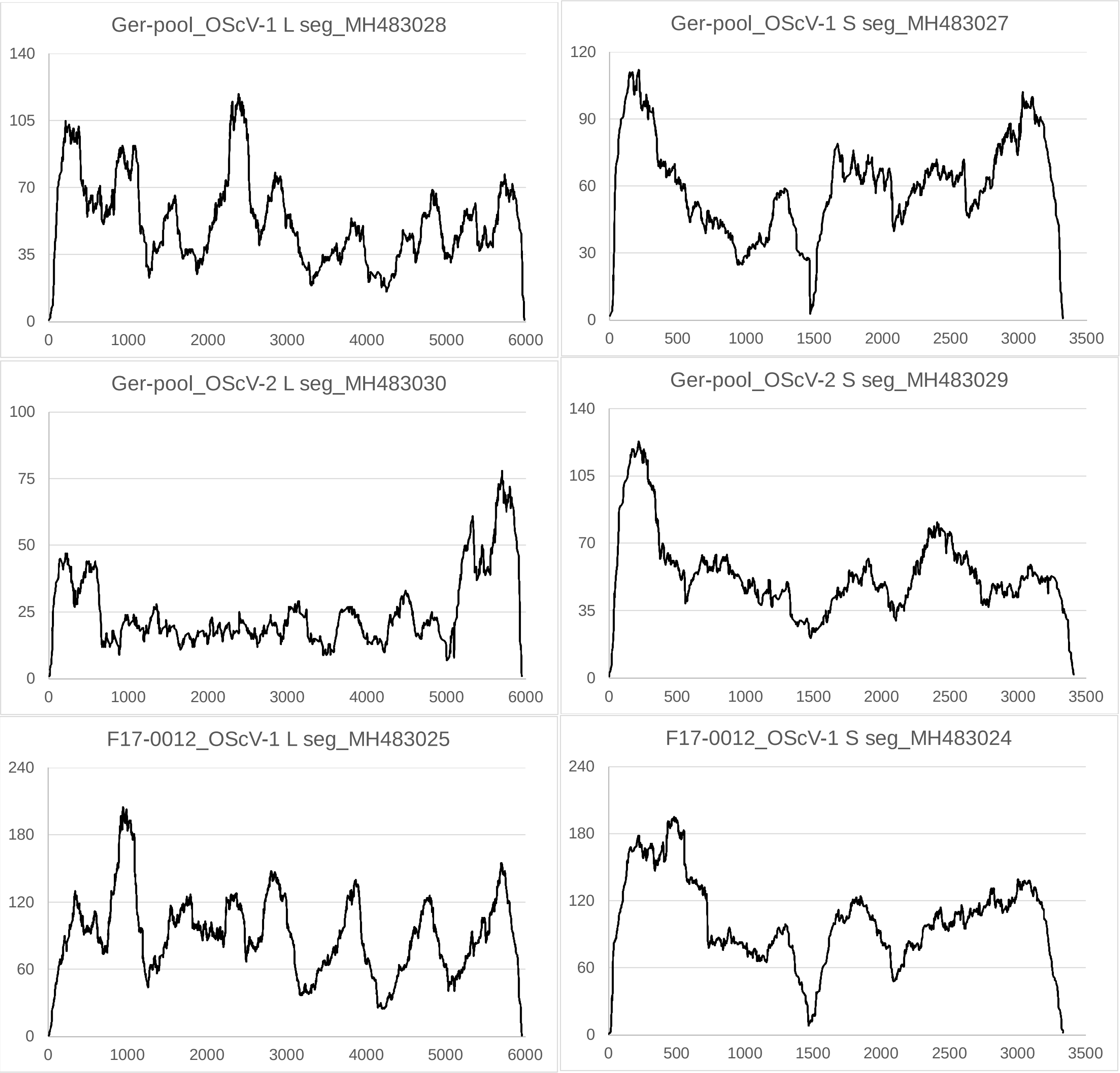

D) Coverage of DaMV-1 L and S segment, snake 4.1 (table 1).

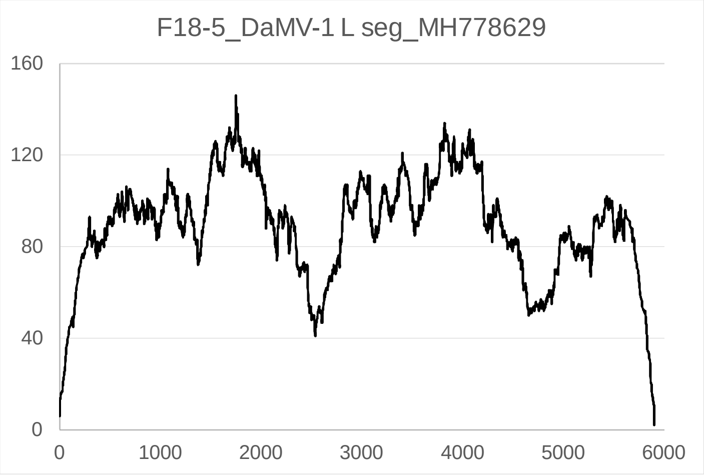

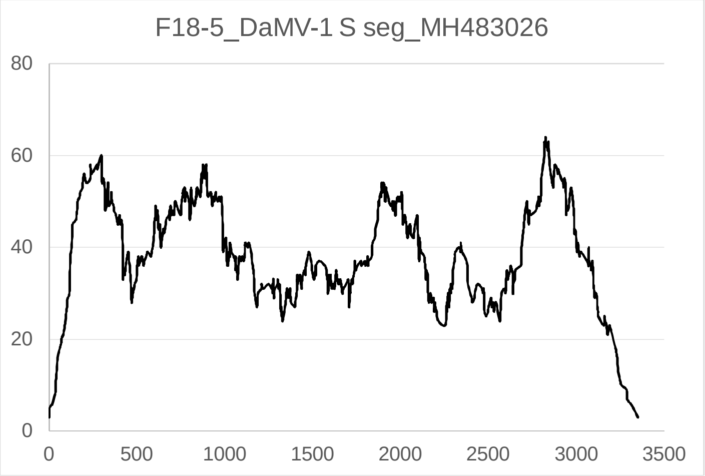

Supplement: S3 Fig — A) Coverage of HISV-1 (pure isolate) and HISV-2, snake 1.4 (Table 1). B) Coverages of VPZV-1 L and S segments, snakes 2.1, 2.2, 2.3, 2.5, 2.6 and 2.7 (Table 1). C) Coverage of OScV-1 and OScV-2 L and S segments, snakes 3.1 and 3.2 (Table 1). D) Coverage of DaMV-1 S segment, snake 4.1 (Table 1). (PDF) [file ppat.1007415.s003.pdf]
