# Supplementary material for: Characterization of Haartman Institute snake virus-1 (HISV-1) and HISV-like viruses—The representatives of genus Hartmanivirus, family Arenaviridae
Source: PLoS Pathog. 2018 Nov 14;14(11):e1007415. doi: 10.1371/journal.ppat.1007415 (PMC6261641; doi:10.1371/journal.ppat.1007415)

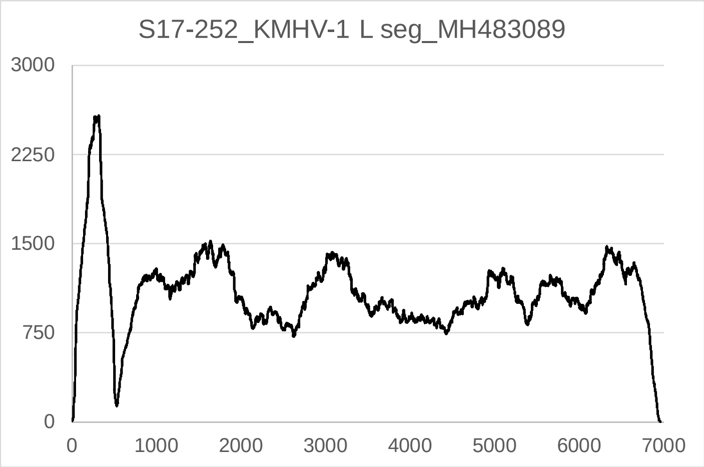
S17-252_KMHV-1 L seg_MH483089
3000
2250
1500
750
0
0 1000 2000 3000 4000 5000 6000 7000

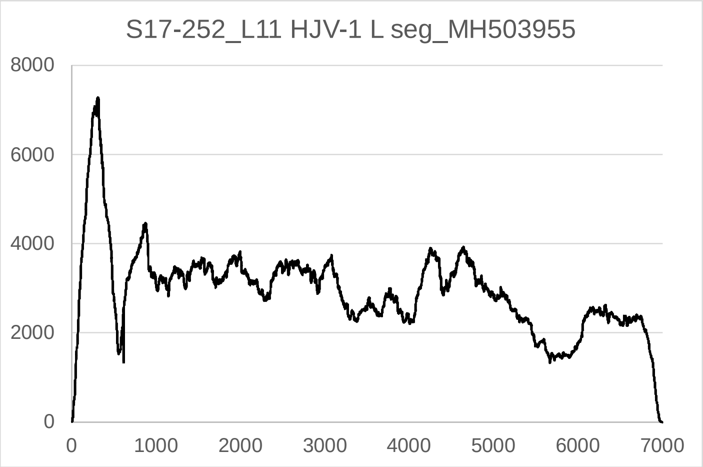
S17-252_L11 HJV-1 L seg_MH503955
8000
6000
4000
2000
0
0 1000 2000 3000 4000 5000 6000 7000

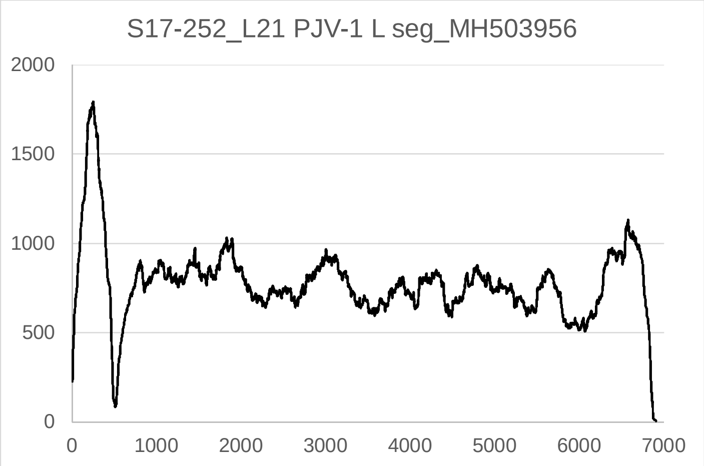
S17-252_L21 PJV-1 L seg_MH503956
2000
1500
1000
500
0
0 1000 2000 3000 4000 5000 6000 7000

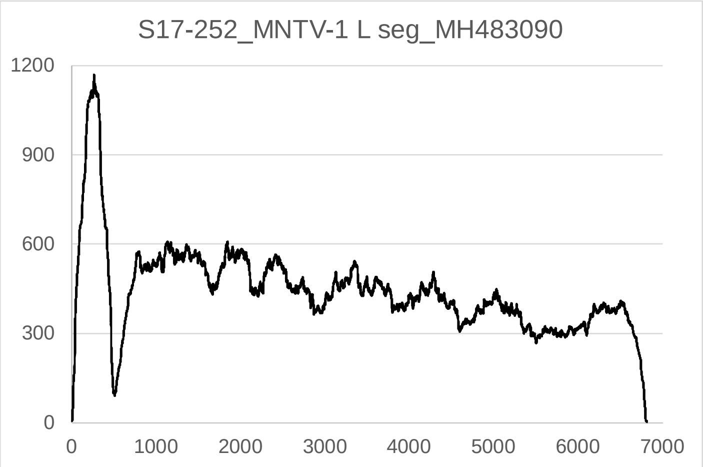
S17-252_MNTV-1 L seg_MH483090
1200
900
600
300
0
0 1000 2000 3000 4000 5000 6000 7000

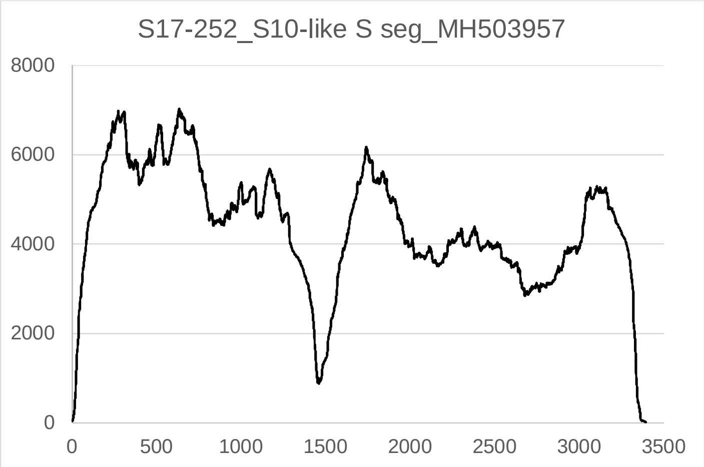
S17-252_S10-like S seg_MH503957
8000
6000
4000
2000
0
0 500 1000 1500 2000 2500 3000 3500

B) Coverage of reptarenavirus L and S segments identified in Snake 2.1 (table 1).

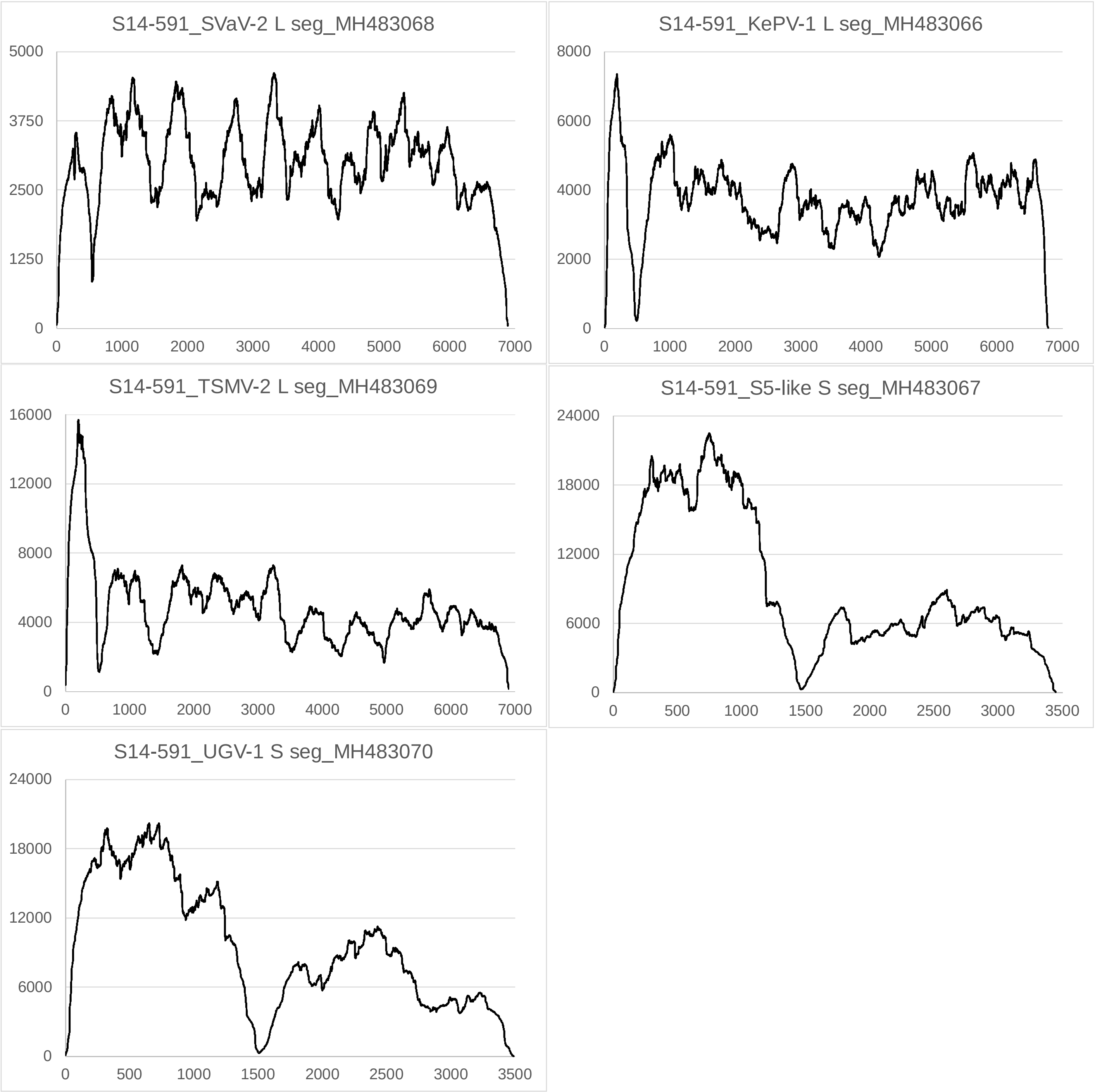

C) Coverage of reptarenavirus L and S segments identified in Snake 2.2 (table 1).

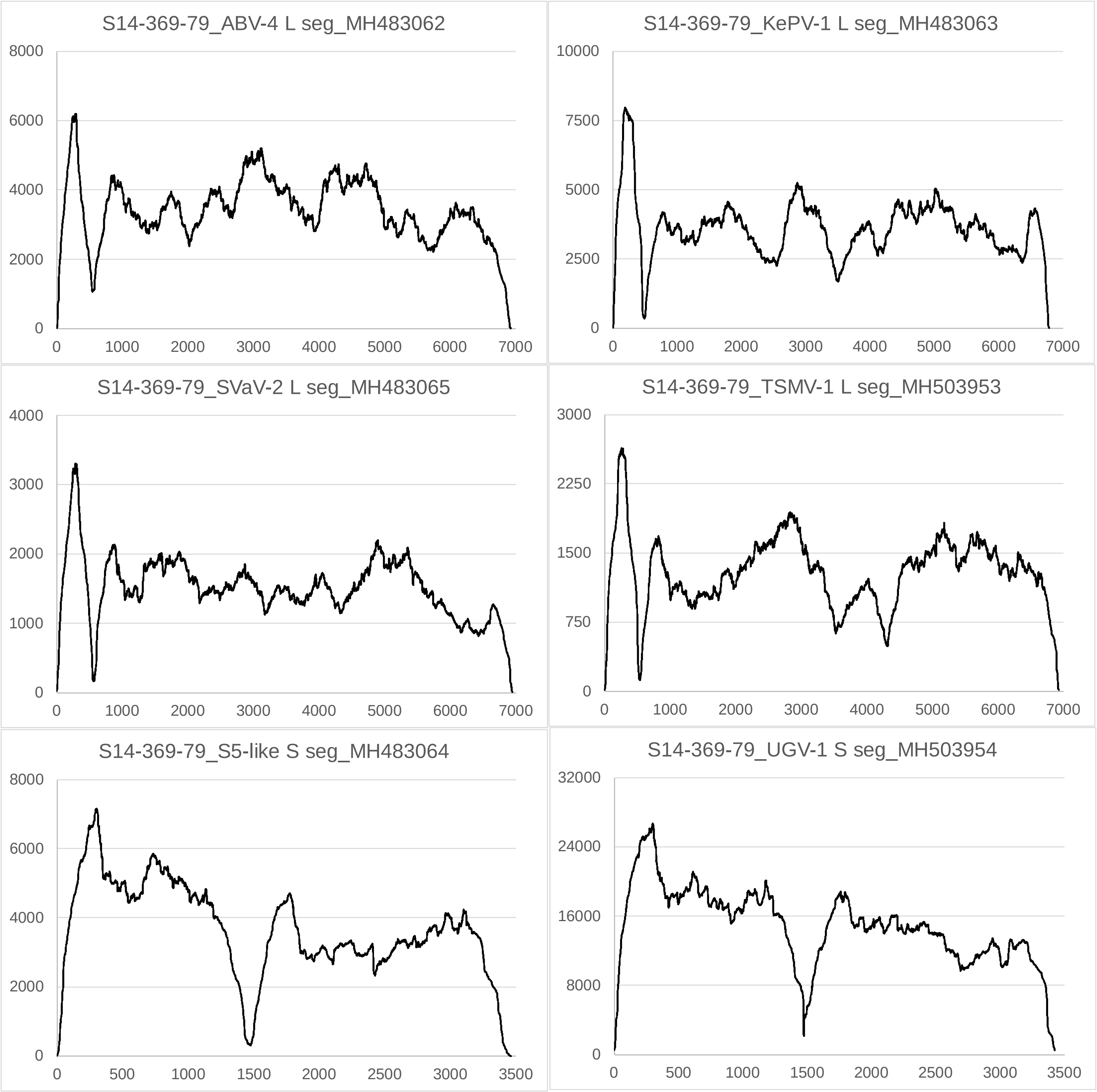

D) Coverage of reptarenavirus L and S segments identified in Snakes 2.3 and 2.4 (table 1).

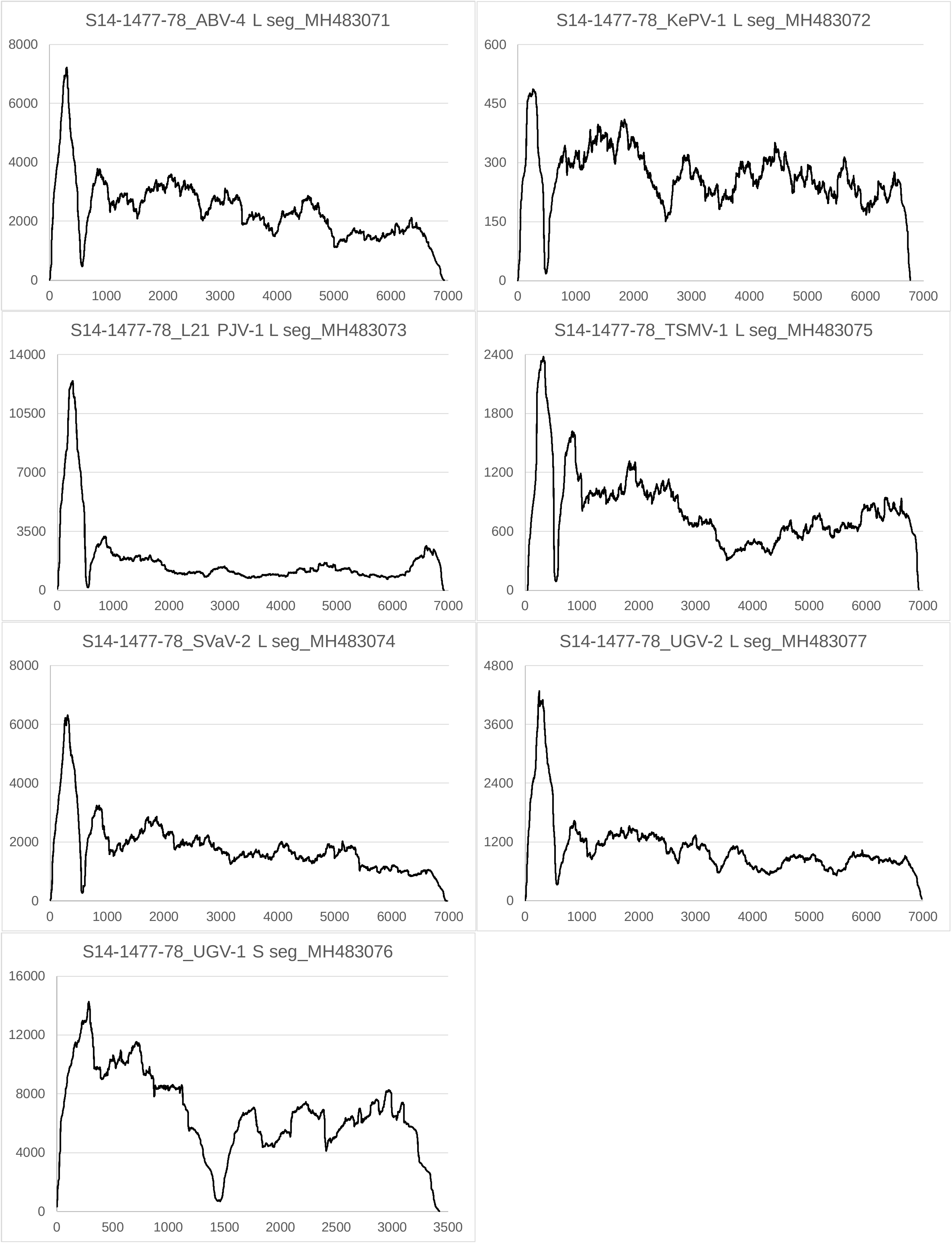

E) Coverage of reptarenavirus L and S segments identified in Snake 2.5 (table 1).

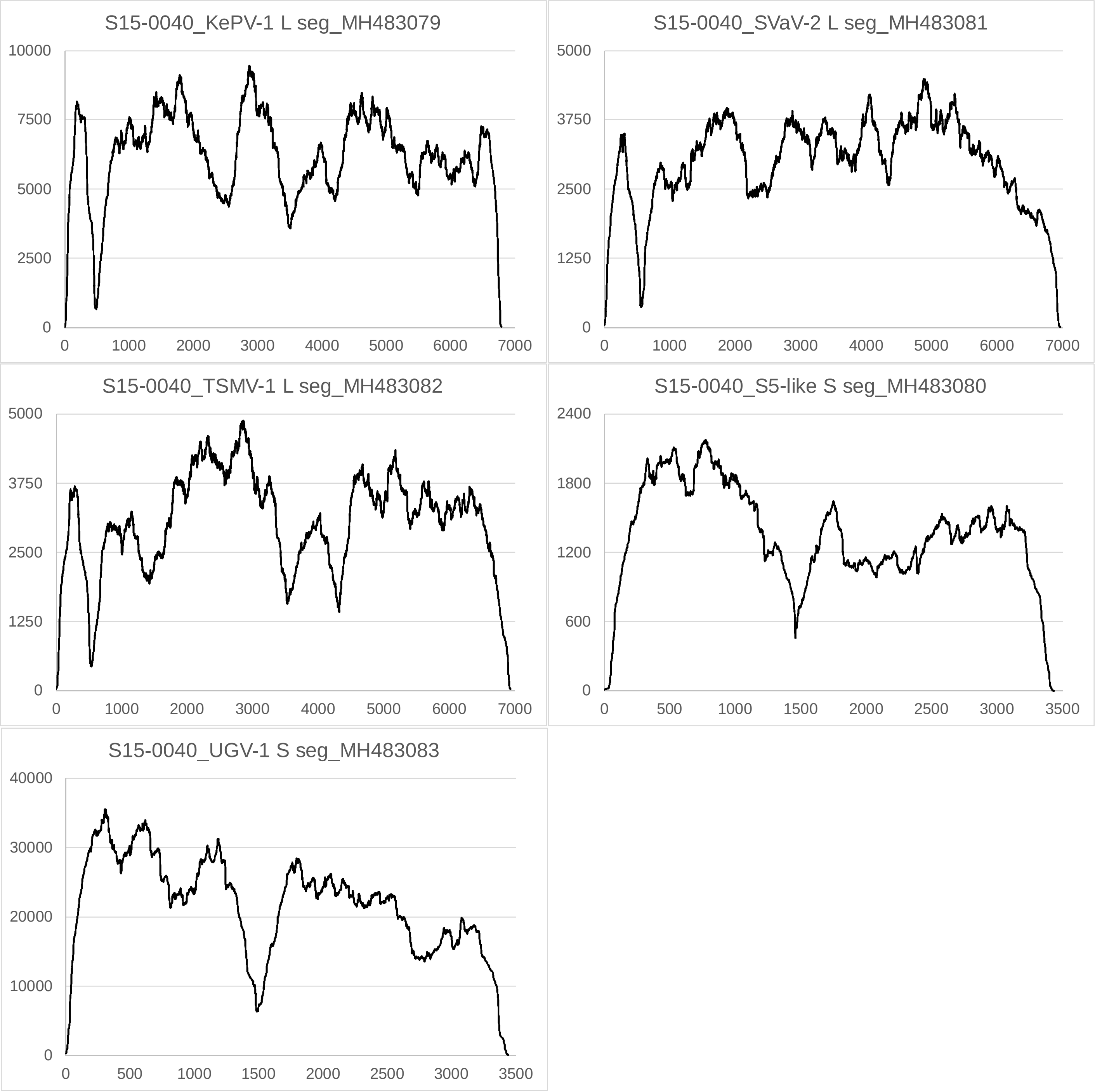

F) Coverage of reptarenavirus L and S segments identified in Snake 2.6 (table 1).

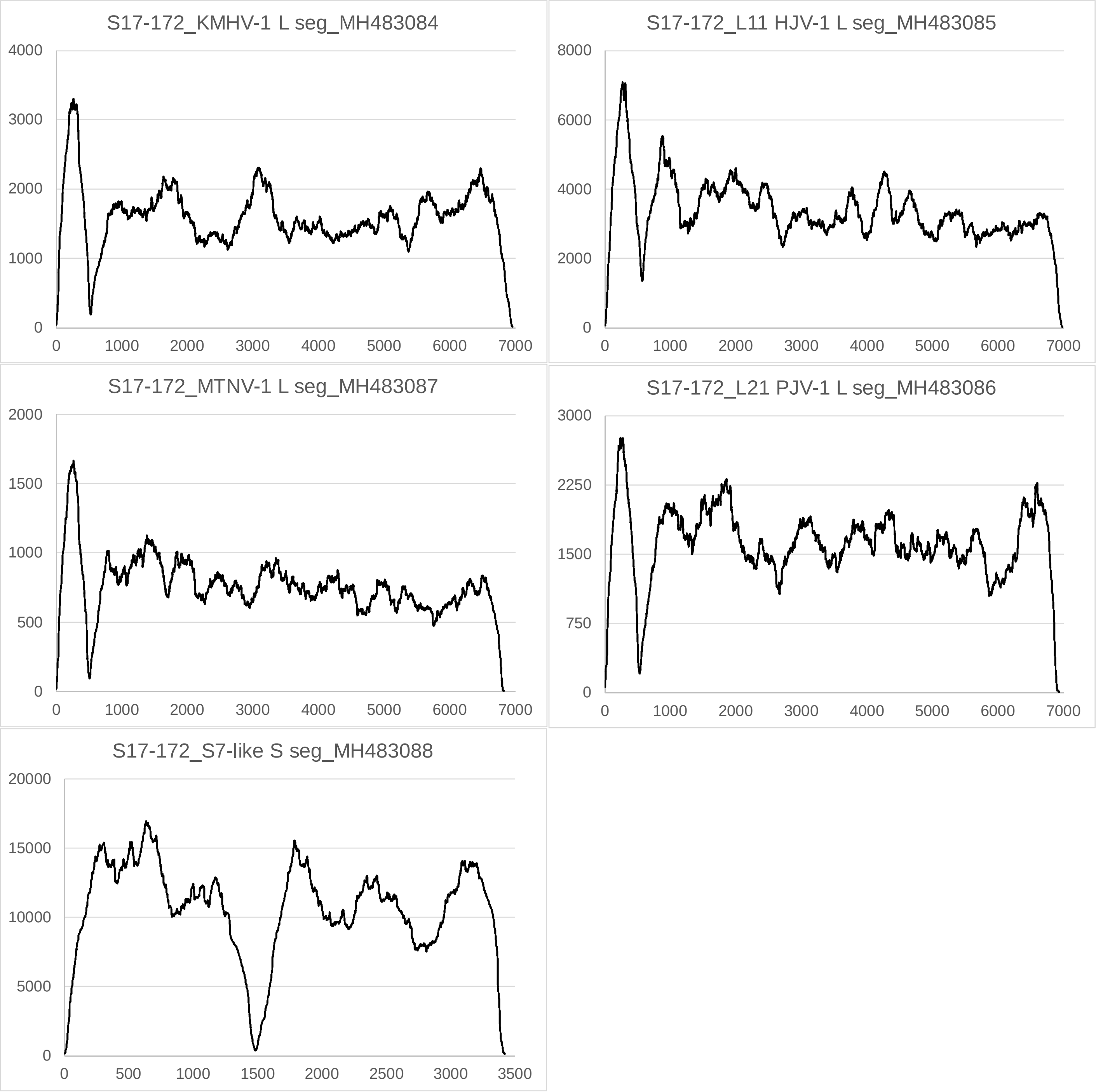

G) Coverage of reptarenavirus L and S segments identified in Snake 2.7 (table 1).

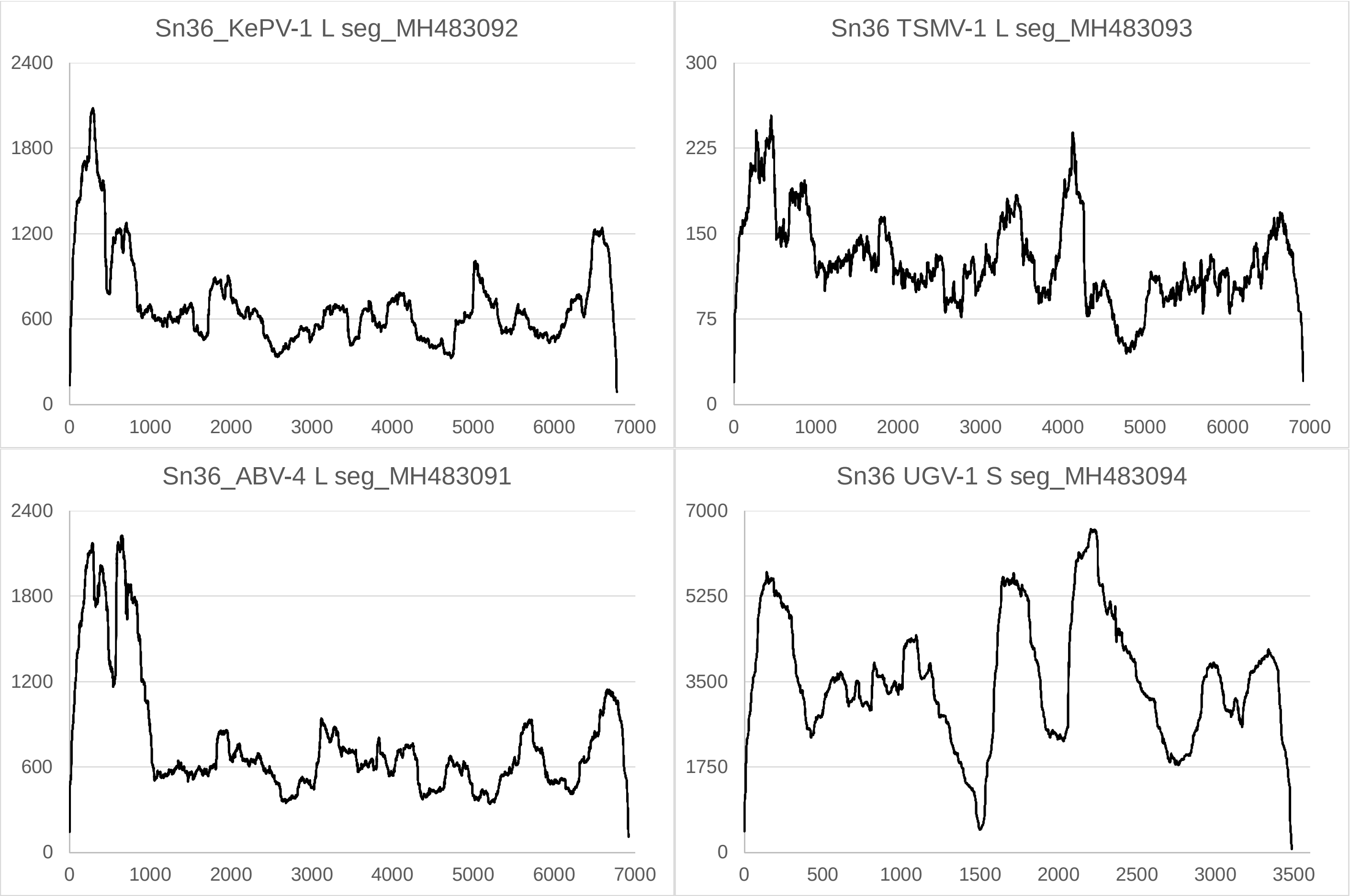

H) Coverage of reptarenavirus L and S segments identified in Snakes 3.1 (table 1).

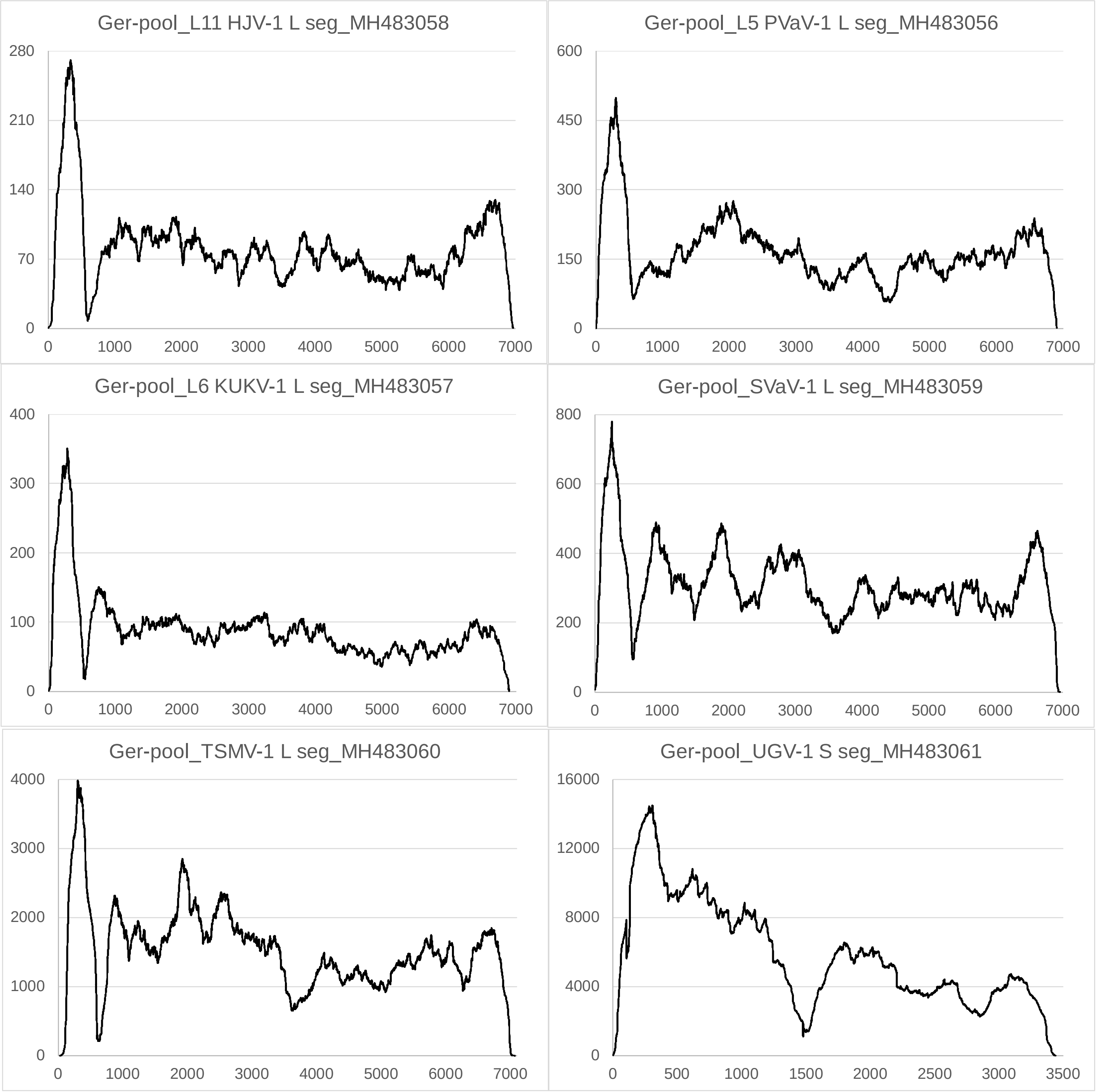

I) Coverage of reptarenavirus L and S segments identified in Snakes 3.2 (table 1).

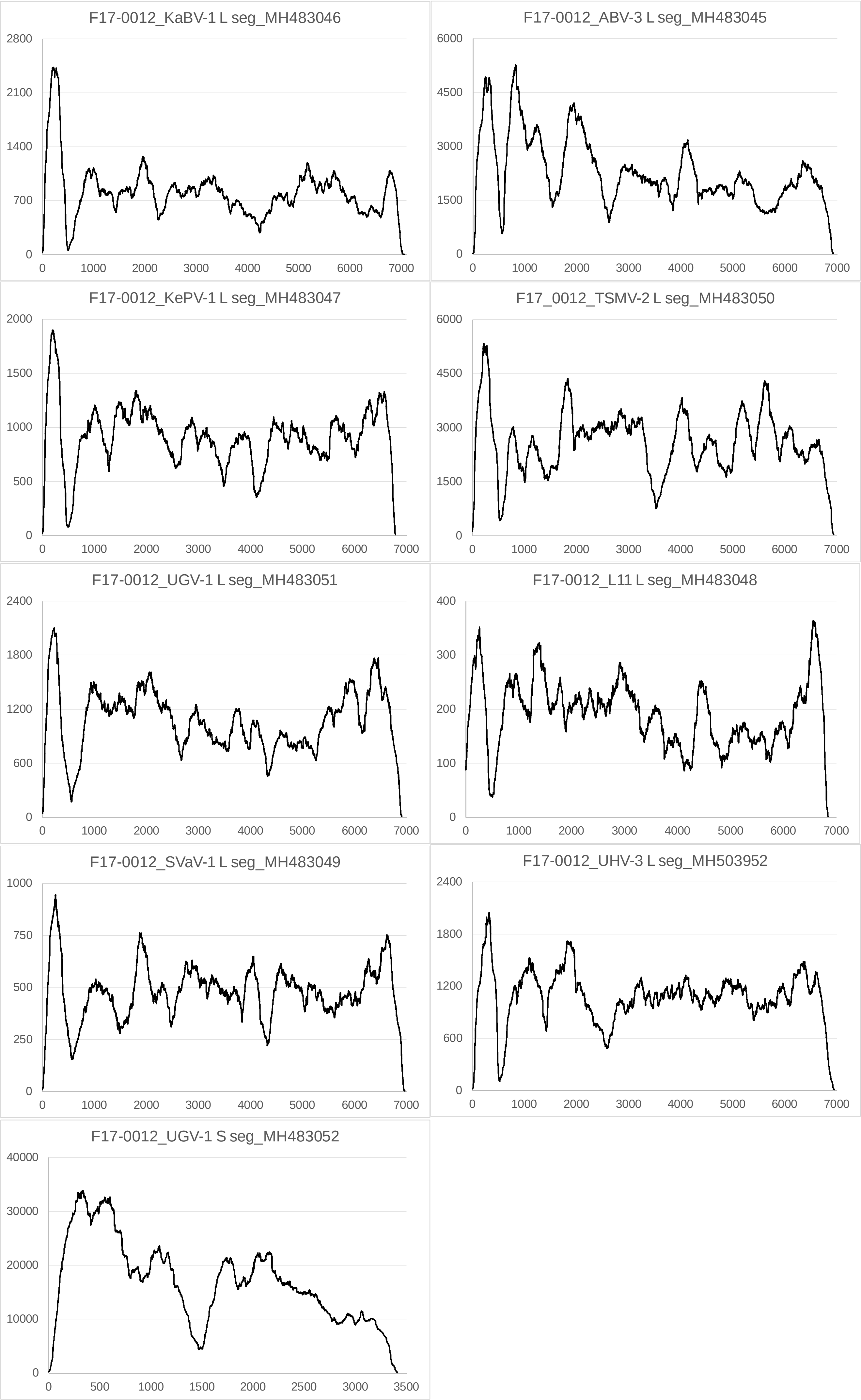

J) Coverage of reptarenavirus L and S segments identified in Snake 4.1 (table 1).

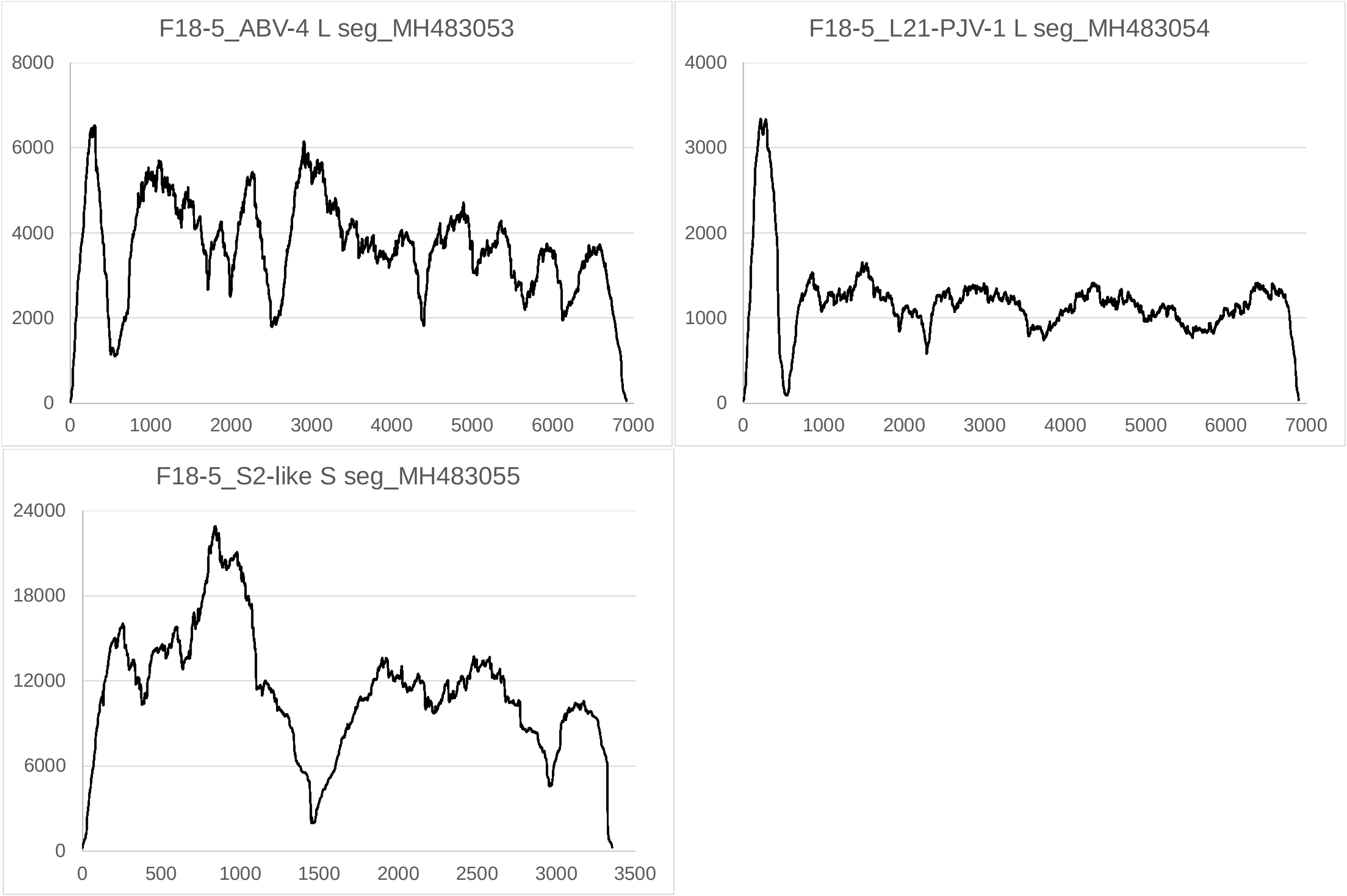

Supplement: S4 Fig — (PDF) [file ppat.1007415.s004.pdf]
