# Supplementary material for: Characterization of Haartman Institute snake virus-1 (HISV-1) and HISV-like viruses—The representatives of genus Hartmanivirus, family Arenaviridae
Source: PLoS Pathog. 2018 Nov 14;14(11):e1007415. doi: 10.1371/journal.ppat.1007415 (PMC6261641; doi:10.1371/journal.ppat.1007415)
Supplement: S1 Table — (PDF) [file ppat.1007415.s005.pdf]

Supplementary table 1. Names, abbreviations, and accession numbers for viruses sequenced in this study.

Genus *Hartmanivirus*

Virus name	Abbreviation	Genome segment	Identifier	Animal ref. in Table 1	GenBank accession no.
Old schoolhouse virus-1	OScV-1	S	F17-0012-1 and -3	3.2. (pool of BIBD pos. snakes)	MH483024
Old schoolhouse virus-1	OScV-1	L	F17-0012-1 and -3	3.2. (pool of BIBD pos. snakes)	MH483025
Dante Muikkunen virus-1	DaMV-1	S	F18-5	4.1.	MH483026
Dante Muikkunen virus-1	DaMV-1	L	F18-5	4.1.	MH778629
Old schoolhouse virus-1	OScV-1	S	Ger-pool	3.1. (pool of BIBD pos. snakes)	MH483027
Old schoolhouse virus-1	OScV-1	L	Ger-pool	3.1. (pool of BIBD pos. snakes)	MH483028
Old schoolhouse virus-2	OScV-2	S	Ger-pool	3.1. (pool of BIBD pos. snakes)	MH483029
Old schoolhouse virus-2	OScV-2	L	Ger-pool	3.1. (pool of BIBD pos. snakes)	MH483030
Haartman Institute Snake virus-1	HISV-1	S	HISV-1 pure isolate	1.1. (origin of isolate)	KR870017
Haartman Institute Snake virus-1	HISV-1	L	HISV-1 pure isolate	1.1. (origin of isolate)	KR870031
Veterinary Pathology Zurich virus-1	VPZV-1	S	S14-369 to -79 pool	2.2. (pool of BIBD pos. snakes)	MH483031
Veterinary Pathology Zurich virus-1	VPZV-1	L	S14-369 to -79 pool	2.2. (pool of BIBD pos. snakes)	MH483032
Veterinary Pathology Zurich virus-1	VPZV-1	S	S14-0591	2.1.	MH483033
Veterinary Pathology Zurich virus-1	VPZV-1	L	S14-0591	2.1.	MH483034
Veterinary Pathology Zurich virus-1	VPZV-1	S	S14-1477 and -1478	2.3. and 2.4 (pool of BIBD pos. snakes)	MH483035
Veterinary Pathology Zurich virus-1	VPZV-1	L	S14-1477 and -1478	2.3. and 2.4 (pool of BIBD pos. snakes)	MH483036
Veterinary Pathology Zurich virus-1	VPZV-1	S	S15-0040	2.5.	MH483037
Veterinary Pathology Zurich virus-1	VPZV-1	L	S15-0040	2.5.	MH483038
Veterinary Pathology Zurich virus-1	VPZV-1	S	S17-172	2.6.	MH483039
Veterinary Pathology Zurich virus-1	VPZV-1	L	S17-172	2.6.	MH483040
Haartman Institute Snake virus-2	HISV-2	S	S17-252	1.4.	MH483041
Haartman Institute Snake virus-2	HISV-2	L	S17-252	1.4.	MH483042
Veterinary Pathology Zurich virus-2	VZVP-2	S	Sn36	2.7. (origin of cell culture grown virus)	MH483043
Veterinary Pathology Zurich virus-2	VZVP-2	L	Sn36	2.7. (origin of cell culture grown virus)	MH483044

Genus *Reptarenavirus*

Virus name	Abbreviation	Genome segment	Identifier	Animal ref. in Table 1	GenBank accession no.
Aurora borealis virus-3	ABV-3	L	F17-0012-1 and -3	3.2. (pool of BIBD pos. snakes)	MH483045
Kaltenbach virus-1	KaBV-1	L	F17-0012-1 and -3	3.2. (pool of BIBD pos. snakes)	MH483046
Keijut pohjoismaissa virus-1	KePV-1	L	F17-0012-1 and -3	3.2. (pool of BIBD pos. snakes)	MH483047
Hipoen jatkoon virus-1	HJV-1	L	F17-0012-1 and -3	3.2. (pool of BIBD pos. snakes)	MH483048
Suri Vanera virus-1	SVaV-1	L	F17-0012-1 and -3	3.2. (pool of BIBD pos. snakes)	MH483049
Tavallinen suomalainen mies virus-2	TSMV-2	L	F17-0012-1 and -3	3.2. (pool of BIBD pos. snakes)	MH483050
University of Giessen virus-1	UGV-1	L	F17-0012-1 and -3	3.2. (pool of BIBD pos. snakes)	MH483051
University of Giessen virus-1	UGV-1	S	F17-0012-1 and -3	3.2. (pool of BIBD pos. snakes)	MH483052
University of Helsinki virus-3	UHV-3	L	F17-0012-1 and -3	3.2. (pool of BIBD pos. snakes)	MH503952
Aurora borealis virus-4	ABV-4	L	F18-5	4.1.	MH483053
Peto jauhoksi virus-1	PJV-1	L	F18-5	4.1.	MH483054
S segment similar to S2	S2-like	S	F18-5	4.1.	MH483055
Peilihimmeli vakooja virus-1	PVaV-1	L	Ger-pool	3.1. (pool of BIBD pos. snakes)	MH483056
Kiva uusi käärme virus-1	KUKV-1	L	Ger-pool	3.1. (pool of BIBD pos. snakes)	MH483057
Hipoen jatkoon virus-1	HJV-1	L	Ger-pool	3.1. (pool of BIBD pos. snakes)	MH483058
Suri Vanera virus-1	SvaV-1	L	Ger-pool	3.1. (pool of BIBD pos. snakes)	MH483059
Tavallinen suomalainen mies virus-1	TSMV-1	L	Ger-pool	3.1. (pool of BIBD pos. snakes)	MH483060
University of Giessen virus-1	UGV-1	S	Ger-pool	3.1. (pool of BIBD pos. snakes)	MH483061
Aurora borealis virus-4	ABV-4	L	S14-369 to -79 pool	2.2. (pool of BIBD pos. snakes)	MH483062
Keijut pohjoismaissa virus-1	KePV-1	L	S14-369 to -79 pool	2.2. (pool of BIBD pos. snakes)	MH483063
S segment similar to S5	S5-like	S	S14-369 to -79 pool	2.2. (pool of BIBD pos. snakes)	MH483064
Suri Vanera virus-2	SVaV-2	L	S14-369 to -79 pool	2.2. (pool of BIBD pos. snakes)	MH483065
Tavallinen suomalainen mies virus-1	TSMV-1	L	S14-369 to -79 pool	2.2. (pool of BIBD pos. snakes)	MH503953
University of Giessen virus-1	UGV-1	S	S14-369 to -79 pool	2.2. (pool of BIBD pos. snakes)	MH503954
Keijut pohjoismaissa virus-1	KePV-1	L	S14-0591	2.1.	MH483066
S segment similar to S5	S5-like	S	S14-0591	2.1.	MH483067
Suri Vanera virus-2	SVaV-2	L	S14-0591	2.1.	MH483068
Tavallinen suomalainen mies virus-2	TSMV-2	L	S14-0591	2.1.	MH483069

University of Giessen virus-1	UGV-1	S	S14-0591	2.1.	MH483070
Aurora borealis virus-4	ABV-4	L	S14-1477 and -1478	2.3. and 2.4 (pool of BIBD pos. snakes)	MH483071
Keijut pohjoismaissa virus-1	KePV-1	L	S14-1477 and -1478	2.3. and 2.4 (pool of BIBD pos. snakes)	MH483072
Peto jauhoksi virus-1	PJV-1	L	S14-1477 and -1478	2.3. and 2.4 (pool of BIBD pos. snakes)	MH483073
Suri Vanera virus-2	SVaV-2	L	S14-1477 and -1478	2.3. and 2.4 (pool of BIBD pos. snakes)	MH483074
Tavallinen suomalainen mies virus-1	TSMV-1	L	S14-1477 and -1478	2.3. and 2.4 (pool of BIBD pos. snakes)	MH483075
University of Giessen virus-1	UGV-1	S	S14-1477 and -1478	2.3. and 2.4 (pool of BIBD pos. snakes)	MH483076
University of Giessen virus-2	UGV-2	L	S14-1477 and -1478	2.3. and 2.4 (pool of BIBD pos. snakes)	MH483077
Aurora borealis virus-4	ABV-4	L	S15-0040	2.5.	MH483078
Keijut pohjoismaissa virus-1	KePV-1	L	S15-0040	2.5.	MH483079
S segment similar to S5	S5-like	S	S15-0040	2.5.	MH483080
Suri Vanera virus-2	SVaV-2	L	S15-0040	2.5.	MH483081
Tavallinen suomalainen mies virus-1	TSMV-1	L	S15-0040	2.5.	MH483082
University of Giessen virus-1	UGV-1	S	S15-0040	2.5.	MH483083
Kuka mitä häh virus-1	KMHV-1	L	S17-172	2.6.	MH483084
Hipoen jatkoon virus-1	HJV-1	L	S17-172	2.6.	MH483085
Peto jauhoksi virus-1	PJV-1	L	S17-172	2.6.	MH483086
Mistä näitä tulee virus-1	MNTV-1	L	S17-172	2.6.	MH483087
S segment similar to S7	S7-like	S	S17-172	2.6.	MH483088
Kuka mitä häh virus-1	KMHV-1	L	S17-252	1.4.	MH483089
Hipoen jatkoon virus-1	HJV-1	L	S17-252	1.4.	MH503955
Peto jauhoksi virus-1	PJV-1	L	S17-252	1.4.	MH503956
Kuka mitä häh virus-1	MNTV-1	L	S17-252	1.4.	MH483090
S segment similar to S10	S10-like	S	S17-252	1.4.	MH503957
Aurora borealis virus-4	ABV-4	L	Sn36	2.7. (origin of cell culture grown virus)	MH483091
Keijut pohjoismaissa virus-1	KePV-1	L	Sn36	2.7. (origin of cell culture grown virus)	MH483092
Tavallinen suomalainen mies virus-1	TSMV-1	L	Sn36	2.7. (origin of cell culture grown virus)	MH483093
University of Giessen virus-1	UGV-1	S	Sn36	2.7. (origin of cell culture grown virus)	MH483094
