# Supplementary material for: Characterization of Haartman Institute snake virus-1 (HISV-1) and HISV-like viruses—The representatives of genus Hartmanivirus, family Arenaviridae
Source: PLoS Pathog. 2018 Nov 14;14(11):e1007415. doi: 10.1371/journal.ppat.1007415 (PMC6261641; doi:10.1371/journal.ppat.1007415)
Supplement: S2 Table — (PDF) [file ppat.1007415.s006.pdf]

Supplementary table 2. Proteomes and amino acid identities between the type species of family *Arenaviridae* genera.

Proteome for the type species of family *Arenaviridae* genera

	Mammarenavirus (LCMV)	Reptarenavirus (GGV)	Hartmanivirus (HISV-1)
GPC	498	427	476
SSP / SP	58	23	55 (37)
myristoylation	yes	no	yes
palmitoylation	no	yes: C10, C19	yes: C34
GP1	207	215	191
GP2	233	189	230
cytoplasmic tail	42	2	48
NP	558	591	582
ZP	90	116	no
myristoylation	yes	no	
TM helix	no	13-31	
RdRp	2210	2066	1958

Amino acid identity based on MAFFT alignment in UGENE

	LCMV-GPC	GGV-GPC	HISV-GPC
LCMV-GPC	100 %	16 %	23 %
GGV-GPC	16 %	100 %	22 %
HISV-GPC	23 %	22 %	100 %

	LCMV-NP	GGV-NP	HISV-NP
LCMV-NP	100 %	32 %	21 %
GGV-NP	32 %	100 %	20 %
HISV-NP	21 %	20 %	100 %

	LCMV-ZP	GGV-ZP
LCMV-ZP	100 %	16 %
GGV-ZP	16 %	100 %

	LCMV-RdRp	GGV-RdRp	HISV-RdRp
LCMV-RdRp	100 %	28 %	21 %
GGV-RdRp	28 %	100 %	20 %
HISV-RdRp	21 %	20 %	100 %
