# Supplementary material for: Characterization of Haartman Institute snake virus-1 (HISV-1) and HISV-like viruses—The representatives of genus Hartmanivirus, family Arenaviridae
Source: PLoS Pathog. 2018 Nov 14;14(11):e1007415. doi: 10.1371/journal.ppat.1007415 (PMC6261641; doi:10.1371/journal.ppat.1007415)

Supplementary table and figure 3. Results of PASC analysis: similarities between hartamanivirus L and S segments above, and top matches for HISV-1 L and S segments below.

L segments

	HISV-1	HISV-2	OScV-1	OScV-2	VPZV-1	VPZV-2	DaMV-1
HISV-1	100,0 %						
HISV-2	88,7 %	100,0 %					
OScV-1	62,8 %	63,4 %	100,0 %				
OScV-2	63,3 %	64,1 %	85,0 %	100,0 %			
VPZV-1	63,9 %	63,4 %	63,3 %	63,9 %	100,0 %		
VPZV-2	64,0 %	62,9 %	62,4 %	63,1 %	85,9 %	100,0 %	
DaMV-1	66,8 %	67,3 %	63,6 %	62,9 %	63,5 %	64,3 %	100,0 %

S segments

	HISV-1	HISV-2	OScV-1	OScV-2	VPZV-1	VPZV-2	DaMV-1
HISV-1	100,0 %						
HISV-2	85,4 %	100,0 %					
OScV-1	59,0 %	58,9 %	100,0 %				
OScV-2	59,0 %	59,3 %	78,0 %	100,0 %			
VPZV-1	59,8 %	59,9 %	58,9 %	57,9 %	100,0 %		
VPZV-2	61,2 %	60,0 %	58,5 %	57,1 %	83,8 %	100,0 %	
DaMV-1	64,9 %	64,3 %	59,5 %	60,4 %	60,7 %	61,0 %	100,0 %

Top matches for lcl|HISV-1_isolate1 L segment:

BLAST-based alignments

1	19,05 %	gi|62766429|gb|AY924389,1| Mammarenavirus|Serra do Navio mammarenavirus
2	18,27 %	gi|821492670|gb|KP071674,1| Reptarenavirus|unclassified Reptarenavirus|unidentified Reptarenavirus
3	18,17 %	gi|34365541|ref|NC_005082,1| Mammarenavirus|Guanarito mammarenavirus
4	16,79 %	gi|164519632|gb|AY216503,2| Mammarenavirus|Bear Canyon mammarenavirus
5	16,44 %	gi|821492083|gb|KP071510,1| Reptarenavirus|unclassified Reptarenavirus|unidentified Reptarenavirus
6	16,38 %	gi|821492546|gb|KP071642,1| Reptarenavirus|unclassified Reptarenavirus|unidentified Reptarenavirus
7	16,21 %	gi|821492209|gb|KP071547,1| Reptarenavirus|unclassified Reptarenavirus|unidentified Reptarenavirus
8	16,20 %	gi|155733526|gb|EF529745,1| Mammarenavirus|Cali mammarenavirus
9	16,10 %	gi|821492247|gb|KP071561,1| Reptarenavirus|unclassified Reptarenavirus|unidentified Reptarenavirus
10	16,09 %	gi|821492286|gb|KP071570,1| Reptarenavirus|unclassified Reptarenavirus|unidentified Reptarenavirus
11	16,03 %	gi|821492697|gb|KP071680,1| Reptarenavirus|unclassified Reptarenavirus|unidentified Reptarenavirus
12	15,91 %	gi|164607196|ref|NC_010250,1| Mammarenavirus|Oliveros mammarenavirus
13	15,91 %	gi|594140721|ref|NC_023763,1| Mammarenavirus|Merino Walk mammarenavirus
14	15,84 %	gi|821492230|gb|KP071552,1| Reptarenavirus|unclassified Reptarenavirus|unidentified Reptarenavirus
15	15,75 %	gi|385140456|gb|JF912084,1| Mammarenavirus|Lymphocytic choriomeningitis mammarenavirus

Top matches for lcl|HISV-1_isolate1 S segment :

BLAST-based alignments

1	18.21%	gi|695315947|gb|KM822120.1| Mammarenavirus|Lassa mammarenavirus
2	17.55%	gi|695313614|gb|KM821828.1| Mammarenavirus|Lassa mammarenavirus
3	17.28%	gi|695314110|gb|KM821882.1| Mammarenavirus|Lassa mammarenavirus
4	17.25%	gi|570339355|gb|KC669699.1| Mammarenavirus|Loei River mammarenavirus
5	17.08%	gi|695315877|gb|KM822113.1| Mammarenavirus|Lassa mammarenavirus
6	16.99%	gi|695313728|gb|KM821841.1| Mammarenavirus|Lassa mammarenavirus
7	16.99%	gi|52627079|ref|NC_006317.1| Mammarenavirus|Brazilian mammarenavirus
8	16.74%	gi|695313567|gb|KM821822.1| Mammarenavirus|Lassa mammarenavirus
9	16.72%	gi|1032562881|gb|KT992420.1| Mammarenavirus|Lassa mammarenavirus
10	16.67%	gi|695313258|gb|KM821783.1| Mammarenavirus|Lassa mammarenavirus
11	16.58%	gi|695313871|gb|KM821856.1| Mammarenavirus|Lassa mammarenavirus
12	16.3%	gi|695313805|gb|KM821848.1| Mammarenavirus|Lassa mammarenavirus
13	16.11%	gi|695313599|gb|KM821826.1| Mammarenavirus|Lassa mammarenavirus
14	15.94%	gi|297381012|gb|GU830839.1| Mammarenavirus|Lassa mammarenavirus
15	15.89%	gi|46373061|gb|AF246121.2| Mammarenavirus|Lassa mammarenavirus

Graphical display of PASC analysis results: top panels show PASC analysis of all hartmanivirus S and L segments; bottom panels show PASC analysis of HISV-1 S and L segments below.

PASC analysis of HISV-1 S (top) and L (bottom) segments.

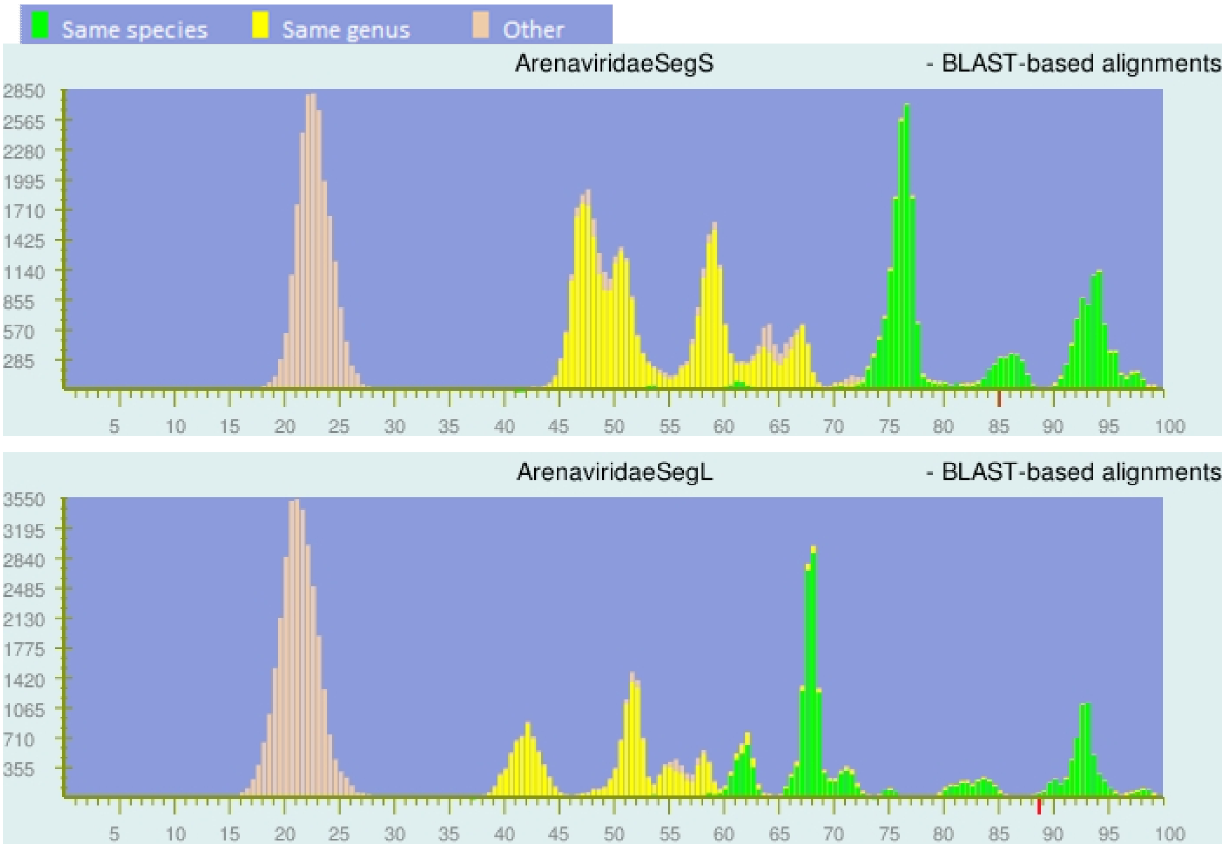

PASC analysis of S (top) and L (bottom) segments of hartmaniviruses described in this study,.

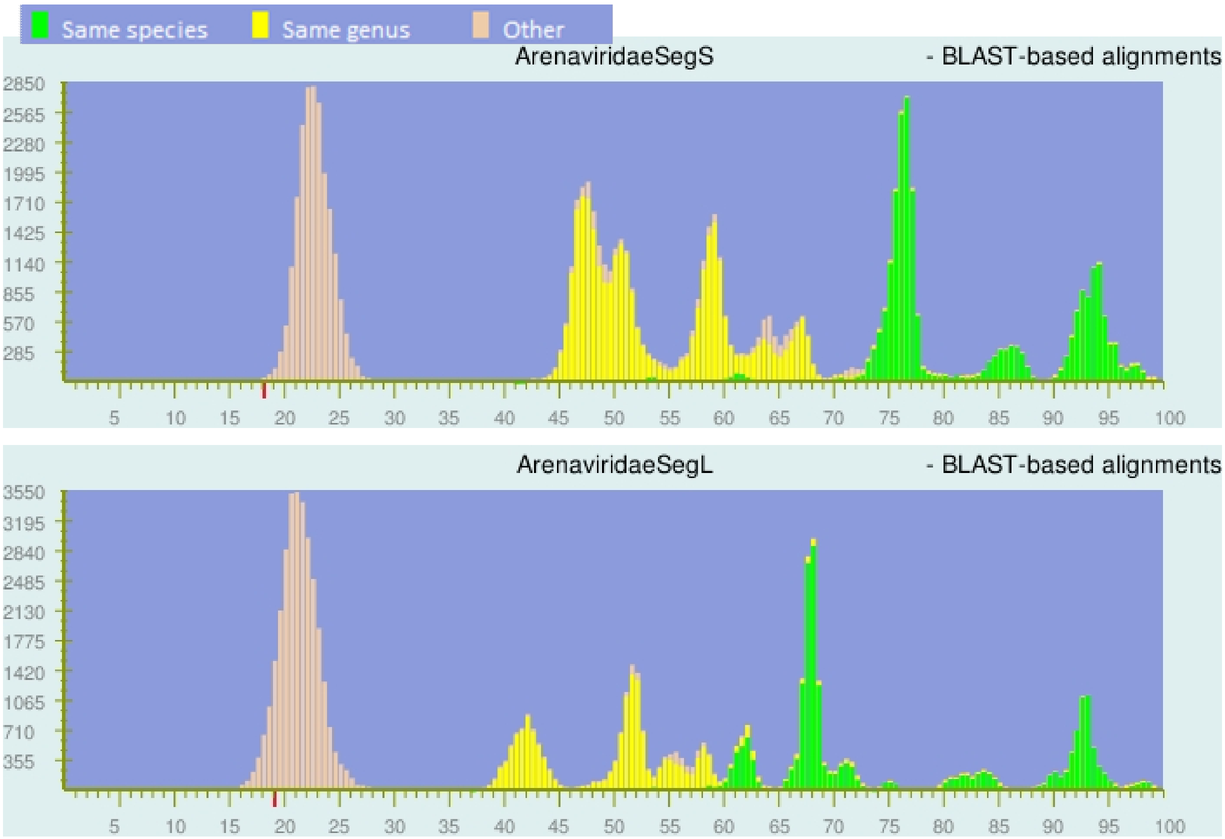

Supplement: S3 Table — (PDF) [file ppat.1007415.s007.pdf]
